# Sequential Supercritical CO_2_ and Subcritical Water Extraction for the Valorisation of Pomegranate (*Punica granatum* L.) By-Products: A Response Surface Methodology Approach

**DOI:** 10.3390/plants15060951

**Published:** 2026-03-19

**Authors:** Miriana Durante, Riccardo Tornese, Rocco Placì, Anna Montefusco, Fabrizio Barozzi, Anna Eleonora Caprifico, Gian-Pietro Di Sansebastiano, Monica De Caroli, Marcello Salvatore Lenucci

**Affiliations:** 1 Istituto di Scienze Delle Produzioni Alimentari (ISPA)-CNR (Consiglio Nazionale delle Ricerche) Via Prov.le Lecce-Monteroni, 73100 Lecce, Italy; miriana.durante@cnr.it; 2 Dipartimento di Scienze e Tecnologie Biologiche ed Ambientali (DiSTeBA), Università del Salento, Via Prov.le Lecce Monteroni, 73100 Lecce, Italy; riccardo.tornese1@unisalento.it (R.T.); rocco.placi@unisalento.it (R.P.); anna.montefusco@unisalento.it (A.M.); fabrizio.barozzi@unisalento.it (F.B.); gp.disansebastiano@unisalento.it (G.-P.D.S.); monica.decaroli@unisalento.it (M.D.C.); 3 School of Allied Health Sciences, Faculty of Health and Life Sciences, De Montfort University, The Gateway, Leicester LE1 9BH, UK; anna.caprifico@dmu.ac.uk

**Keywords:** agro-industrial by-products, bioactive compounds, biorefinery approach, circular economy, critical fluids, green extraction, maltodextrins, extract cytotoxicity

## Abstract

Pomegranate marc is a major, underutilized juice industry by-product rich in lipophilic polyunsaturated fatty acids—notably conjugated α-linolenic acids (CLnAs)—and hydrophilic polyphenols with potent antioxidant and anti-inflammatory properties. Despite its potential for nutraceutical, cosmetic, and pharmaceutical applications, this matrix remains largely unexploited. This study presents a novel, sequential in-line extraction strategy combining supercritical CO_2_ (ScCO_2_) and subcritical water (scW) to recover complementary bioactive fractions. Both extraction steps were optimized via Response Surface Methodology (RSM). Box–Behnken optimization of ScCO_2_ (43 MPa, 76 °C, 6.4 L min^−1^, 124 min) yielded 30 g kg^−1^ dry weight (dw) of oleoresin, achieving a 68% recovery of total oil. Subsequent scW extraction was optimized at 149 °C, with a 40 L kg^−1^ water-to-solute ratio and 73 min extraction time, yielding 47 g kg^−1^ dw of total phenolics (58% recovery). Strong agreement between experimental and predicted values confirmed the robustness of the models. Comprehensive profiling revealed a diverse phytocomplex including fatty acids, tocopherols, flavonoids, soluble sugars, and polysaccharides. Antioxidant assays confirmed that both γ-tocopherol and polyphenols significantly contribute to the extracts’ bioactivity. To improve physical handling, the aqueous fractions were converted into solid dispersions via spray drying with maltodextrin. Preliminary in vitro biological assessments on HEK-293 (human embryonic kidney) and MCF-7 (Michigan Cancer Foundation-7) cell lines suggested that the maltodextrin-based formulations may modulate the cytotoxic profile compared to the free extract, with exploratory results showing dosage-dependent variations in cell viability across the two lines. This work suggests a potentially scalable and sustainable biorefinery approach for the integral valorisation of pomegranate marc, offering a basis for a pathway to produce solvent-free bioactives.

## 1. Introduction

Pomegranate (*Punica granatum* L.), a cornerstone of ancient horticulture dating back to 3000 BC, has transcended its historical status to become a globally significant “superfood,” valued for its unique nutritional density and therapeutic versatility [1]. This biological relevance is intrinsically linked to the plant’s ability to synthesize a wide array of specialized secondary metabolites, which define the unique botanical fingerprint of the species. While Iran, India, and China dominate global production, cultivation has surged across the Mediterranean basin, particularly in Italy. In regions such as Sicily, Puglia, Calabria, and Sardinia, production now exceeds 30,000 tonnes annually across 1900 hectares. However, this domestic output remains insufficient to satisfy rising consumer demand, necessitating substantial imports from Turkey, Israel, and Spain [2,3].

The pharmacological prestige of the fruit is notably attributed to its potent polyphenolic matrix—dominated by ellagitannins such as punicalagin and ellagic acid—as well as essential vitamins C and K [4,5]. These compounds exert systemic health benefits, ranging from metabolic and cardiovascular regulation to dermatological protection [6,7]. Recent evidence has further highlighted the pro-angiogenic potential of pomegranate extracts, suggesting a role in vascular regeneration and accelerated tissue repair [8,9]. Ellagitannins derive from the shikimate and phenylpropanoid routes through the oxidation and polymerization of hexahydroxydiphenoyl esters, ultimately forming punicalagin, the predominant hydrolysable tannin in pomegranate. Flavonoids, including anthocyanins and flavonols, derive from the chalcone branch and contribute to pigmentation, UV protection, and redox regulation. These metabolites accumulate mainly in the peel and internal membranes, where they act as chemical barriers against biotic and abiotic stress. This distribution explains the higher phytochemical content of non-edible fractions compared to arils.

While the edible arils are the primary focus of consumption, the entire botanical structure—including bark, flowers, and leaves—serves as a rich reservoir of bioactive phytochemicals [1].

**Figure 1:**
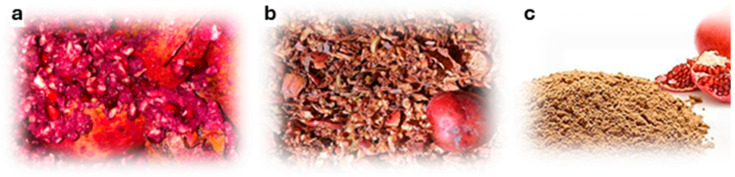
Fresh (**a**) and dehydrated pomegranate marc (**b**), marc powder (**c**).

The industrial transformation of pomegranate, primarily for juice production, generates a massive volume of agro-industrial residues, collectively termed “marc” (Figure 1). This fraction comprises the rind, albedo (spongy mesocarp), carpel membranes, and kernels. Together, these components account for approximately 54% of the total fruit mass—equivalent to roughly 1.62 million tons of annual waste globally [10,11]. Despite being rich in high-value components such as pectin, lignin, and ellagitannins, these residues are frequently discarded or diverted to low-value streams like composting or biogas [12,13]. Pomegranate peels alone contain over 48 distinct bioactive compounds with proven antimicrobial, antidiabetic, and neuroprotective properties, offering a significant opportunity for upcycling into natural preservatives and functional ingredients [5,14,15]. Furthermore, the lipid fraction within the kernels is uniquely rich in punicic acid (up to 83.5%), a conjugated linolenic acid sought after for its potent antioxidant and anti-carcinogenic applications [16,17].

To transition toward a circular bioeconomy, the development of efficient biorefinery strategies is essential. While traditional extraction techniques (e.g., Soxhlet, maceration) are well-established, they are often hindered by long processing times, high solvent consumption, and the potential degradation of thermolabile compounds [18,19]. Consequently, “green” extraction technologies—including ultrasound, microwave, and pulsed electric fields—have emerged as sustainable alternatives [20,21,22]. Among these, supercritical fluid extraction using CO_2_ (ScCO_2_) and subcritical water extraction (scW) are particularly advantageous due to their high selectivity, lack of toxic residues, and minimal environmental footprint. Despite their potential, the industrial adoption of these technologies is often limited by operational complexity and the need for matrix-specific optimization [23]. Unlike previous studies that investigated ScCO_2_ or subcritical water extraction independently, this work integrates both technologies in a single sequential in-line process. This strategy enables the stepwise recovery of lipophilic and hydrophilic bioactive fractions from the same biomass, improving overall valorisation efficiency while maintaining a solvent-free green extraction workflow.

Recent literature has demonstrated the efficacy of these fluids in valorising pomegranate fractions. Rivas et al. [24] successfully employed Response Surface Methodology (RSM) to optimize ScCO_2_ parameters, maximizing the recovery of antioxidants from pomegranate peels. Similarly, Natolino and Da Porto [25] provided critical insights into the extraction of seed oil, elucidating the complex solubility and mass transfer dynamics inherent in the process. Notably, Tan et al. [26] demonstrated that the co-processing of peels and seeds can enhance extraction efficiency, suggesting that matrix synergies between different fruit components may facilitate the release of bioactives.

Building on this foundation, our study proposes for the first time a sequential, in-line green extraction strategy to fully valorise pomegranate marc without the use of harmful organic solvents. The process integrates ScCO_2_ and scW in tandem as a continuous biorefinery chain, enabling the sequential recovery of different classes of bioactive compounds. This approach allows the selectively extraction of lipophilic compounds from seeds and hydrophilic compounds from peels—including ellagic acid, punicic acid, and pectin—thereby maximizing biomass utilization and distinguishing this integrated approach from independent extraction methods [27,28]. Furthermore, to facilitate the industrial application of the scW extracts, we investigate the incorporation of the aqueous fractions into a maltodextrin matrix. Maltodextrins are widely used as carriers in spray drying and facilitate the conversion of liquid extracts into solid dispersions. This improves the physical handling, flowability, and dosage standardization of the resulting powders [29,30,31,32]. This formulation strategy is aimed at producing manageable, free-flowing ingredients suitable for the nutraceutical and cosmetic industries.

Finally, the study includes preliminary in vitro assessments using human embryonic kidney (HEK-293) and breast adenocarcinoma (MCF-7) cell lines. These exploratory screenings were designed to evaluate the cytotoxic potential of the pomegranate phytocomplex and to observe whether its formulation within a carbohydrate matrix influences cellular viability compared to the free extract. This integrated approach aims to provide an initial screening of the safety and biological activity of the recovered fractions, establishing a foundation for future functional studies.

## 2. Results and Discussion

### 2.1. Optimization of ScCO_2_ Extraction

The recovery of oleoresin from pomegranate marc via ScCO_2_ depends on pressure, temperature, CO_2_ flow rate, and extraction time. To systematically evaluate these effects, a Box–Behnken experimental design (BBD) was employed. The experimental yields (expressed as g of extract per 100 g of dry matrix) exhibited a broad range—from a minimum of 5.8% to near-exhaustive recovery—depending on the specific configuration of pressure (X_1_), temperature (X_2_), CO_2_ flow rate (X_3_), and time (X_4_).

To identify the most accurate correlation between these variables and the extraction yield, four semi-empirical models—linear, two-factor interaction (2FI), quadratic, and cubic—were statistically compared (Table 1).

As shown in the initial Analysis of Variance (ANOVA), both linear and quadratic models reached statistical significance (*p* < 0.001). However, the quadratic model provided a superior representation of the system.

This model was characterized by a robust coefficient of determination (R^2^ = 0.8130) and a non-significant lack of fit (*p* = 0.4994). These results suggest that the observed data points were well-aligned with the predicted response surface. Moreover, the close agreement between R^2^ adj (0.6940) and R^2^ pred (0.3501) indicates acceptable predictive consistency and confirms that the quadratic model is not overfitted within the investigated design space. Preliminary regression analysis indicated that temperature (X_2_), CO_2_ flux (X_3_), and time (X_4_) were the primary linear drivers of yield, while the quadratic terms for these factors further underscored the non-linear complexity of the extraction kinetics.

Predictive robustness was enhanced and model complexity reduced by constructing a reduced quadratic model in which non-significant terms (*p* > 0.1) were eliminated while preserving model hierarchy. This refinement was supported by improvements in both the Akaike Information Criterion and the Bayesian Information Criterion. As a result, the signal-to-noise ratio increased substantially, with the Adequacy of Precision rising from 12.31 to 16.82 (Table 2). The predicted R^2^ (0.5891) showed acceptable agreement with the adjusted R^2^ (0.7331), as the difference is less than 0.2, confirming the adequate predictive capability of the model. This value is consistent with the inherent experimental noise of supercritical extractions performed on heterogeneous botanical composites (seeds, peel, and albedo). The final model accounted for 78.5% of the variability in oleoresin yield with a low coefficient of variation (C.V. = 10.31%), confirming its suitability for laboratory-scale process optimization and providing baseline data for potential future scale-up. The resulting second-order polynomial equations for predicting yield (Y_1_) in terms of coded (1) and actual (2) variables are presented below:

(1) $$\ln\left(Y_{1}\right)=3.48+0.21X_{1}+0.54X_{2}+0.34X_{3}+0.29X_{4}+0.36X_{2}^{2}-0.44X_{3}^{2}-0.28X_{4}^{2}$$

(2) $$\ln\left(Y_{1}\right)=1.292+0.014X_{1}-0.081X_{2}+0.412X_{3}+0.023X_{4}+0.001X_{2}^{2}-0.027X_{3}^{2}$$

**Figure 2:**
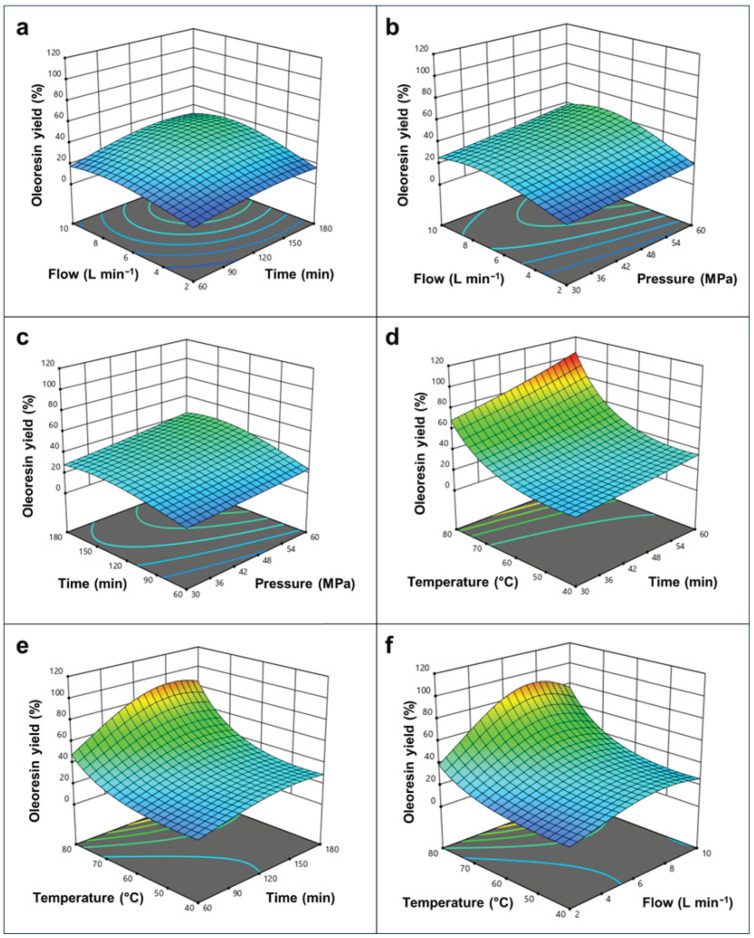
Three-dimensional (3D) response surface plots illustrating the interactive effects of ScCO_2_ process parameters on the oleoresin extraction yield (%) from the raw pomegranate matrix. Panels (**a**–**f**) represent the different binary combinations of independent variables (flow rate, pressure, temperature, and time). Each plot was generated by varying two factors within their experimental range while maintaining the remaining two variables constant at their respective central levels.

The interactive effects between these parameters are visually elucidated in the 3D response surface plots (Figure 2).

The response surfaces showed that pressure and temperature positively affected the ScCO_2_ extraction yield, confirming their role in enhancing solvent strength and mass transfer. A slight efficiency decrease was observed at CO_2_ flow rates above 7.6 L min^−1^, likely due to reduced residence time, while extraction reached a plateau after approximately 150 min, indicating depletion of readily accessible lipophilic components. Computational optimization was subsequently performed to define the conditions that maximize oleoresin yield while minimizing the intensity of the operating parameters—a step towards enhancing process sustainability (Table 3).

The highest desirability score (0.706) was achieved at 43 MPa, 76 °C, and 6.4 L min^−1^ for 124 min, yielding a predicted recovery of approximately 66.5% (Table 4).

These optimal conditions differ notably from those reported in the previous literature, such as Rivas et al. [24] (30 MPa, 55 °C), Natolino and Da Porto [25] (32 MPa, 60 °C), and Tan et al. [26] (40 MPa, 60 °C). These discrepancies are primarily due to the heterogeneity of the starting material. Unlike studies focused on isolated peel or seeds, our work employs whole pomegranate marc (3:1 peel/seed). The presence of spongy albedo and lipid–protein associations increases internal mass transfer resistance. Consequently, a more intense pressure–temperature regime is required to achieve efficient valorisation, highlighting the need to tailor operating parameters to the specific by-product.

### 2.2. Optimization of scW Extraction

Following the exhaustive recovery of the lipophilic fraction, the remaining delipidated pomegranate marc was subjected to scW to isolate hydrophilic bioactive components, primarily phenolic compounds. This sequential approach exploits the tunable polarity of subcritical water, comparable to common organic solvents, while preserving a green processing profile. The optimization of the scW process was conducted using the same Box–Behnken experimental framework applied previously, evaluating the impact of temperature ($X_{1}$), water-to-solute ratio ($X_{2}$), and extraction time ($X_{3}$) on the total phenolic yield ($Y_{2}$). Statistical evaluation of the experimental data, as detailed in Table 5, revealed that the linear model provided the most accurate representation of the extraction process (*p* = 0.0002, *F*-value = 12.57).

Unlike the ScCO_2_ stage, which required a quadratic fit to capture complex interactions, the scW phenolic recovery within the investigated domain followed a largely first-order relationship. This linear behaviour is consistent with the 100–160 °C range, where water’s properties change quasi-linearly and drive phenolic solubilization [33]. The model demonstrated high reliability, with an R^2^ of 0.7154 and an adjusted R^2^ of 0.6585 that aligned reasonably with the predicted R^2^ (0.5004), indicating satisfactory agreement between fitting and predictive performance. The small difference between R^2^adj and R^2^pred confirms that the model is not overfitted and retains adequate predictive capability within the design space. In addition, the non-significant lack of fit (*p* = 0.3390) and the strong signal-to-noise ratio (Adeq. Prec. = 11.577) further support the suitability of the linear model for describing the influence of the operating parameters. ANOVA results pinpointed temperature ($X_{1}$) and the water-to-solute ratio ($X_{2}$) as the dominant factors driving phenolic solubilization, whereas extraction time ($X_{3}$) did not exert a statistically significant influence within the tested ranges (*p* = 0.1140). This finding is consistent with previous work by Yan et al. [34], who identified temperature as the primary driver of phenolic extraction efficiency using subcritical water, likely due to the significant reduction in water’s dielectric constant and viscosity at elevated temperatures.

The relationship between the variables and the phenolic yield (Y_2_) is mathematically expressed by the following first-order polynomial equations in coded (3) and actual (4) terms:

(3) $$Y_{2}=46.08+12.80X_{1}+8.79X_{2}+4.60X_{3}$$

(4) $$Y_{2}=-29.61+0.43X_{1}+0.44X_{2}+0.10X_{3}$$

**Figure 3:**
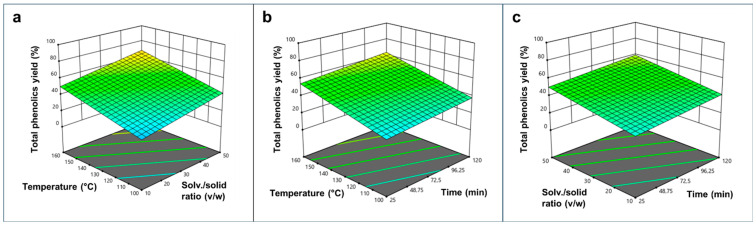
Three-dimensional (3D) response surface plots illustrating the interactive effects of scW process parameters on the phenolics extraction yield (%) from the delipidated pomegranate matrix. Panels (**a**–**c**) represent the different binary combinations of independent variables (temperature, solvent-to-solid ratio, and time). Each plot was generated by varying two variables within the experimental range and keeping the third variable fixed at the central level.

The three-dimensional response surface plots (Figure 3) visually corroborate these findings, showing a steady increase in phenolic recovery as temperature and the solvent-to-solid ratio rise.

To identify a commercially viable process, a multi-objective optimization was performed with the goal of maximizing yield while minimizing thermal and water inputs (Table 6).

Two nearly identical solutions emerged (Table 7) with a desirability score of 0.615. The optimal conditions were established at 149 °C, a water-to-solute ratio of 40 L kg^−1^, and a duration of approximately 73 min, resulting in a predicted phenolic yield of 58.6%.

Interestingly, these results deviate from the findings of Yan et al. [34], who reported a quadratic fit and milder optimal conditions (126.1 °C, 18.5 min, and 54.8 L kg^−1^) for pomegranate peel extraction. This divergence is likely a consequence of our integrated, sequential extraction strategy. The preliminary ScCO_2_ treatment not only removes lipids but also significantly alters the structural integrity and packing density of the marc within the extraction vessel.

This in-line processing likely increases the recalcitrance of the lignocellulosic matrix, necessitating the higher temperatures and longer contact times observed in our study to achieve efficient mass transfer. Furthermore, differences in vessel geometry and heat transfer dynamics between the systems may contribute to the longer residence time required in our setup to reach comparable extraction efficiencies. By tailoring these parameters to the delipidated matrix, we ensure a more exhaustive valorization of the pomegranate marc compared to single-stage water extractions.

### 2.3. Validation of the Optimized Biorefining Process and Mass Balance

The robustness of the predictive models was validated by implementing the optimized sequential biorefining process using the finalized ScCO_2_ and scW parameters (Table 8). This in-line configuration enabled a seamless transition from lipophilic to hydrophilic recovery while reducing handling losses and preserving biomass integrity for subsequent steps.

A comprehensive mass balance (Table 9) confirmed the high efficiency of the integrated workflow. From 200 g of raw marc, the ScCO_2_ step yielded 6.1 ± 0.5 g of oleoresin, corresponding to 3.0% of the biomass and to an extraction efficiency of 68.1% of the available oleoresin (Table 10), in excellent agreement with the predicted 68%. The resulting delipidated matrix (193.6 ± 0.8 g) was processed by scW to obtain 48.8 ± 2.0 g of phenolic-rich extract (24.4% of the raw mass). The final exhausted residue weighed 144.8 ± 2.0 g, and the overall mass balance closed at 99.8%, with only minimal loss attributable to handling or moisture variation.

The quantitative analysis further validated the predictive accuracy of the RSM framework. In the scW step, the recovery of 47.1 ± 6.9 g kg^−1^ of total phenolics corresponded to an extraction efficiency of 58.7%, closely matching the predicted 59% (Table 10). The lower phenolic content of the delipidated matrix (80.3 ± 7.0 g kg^−1^ vs. 98.0 ± 8.8 g kg^−1^ in raw marc) suggests partial degradation or transformation of thermolabile compounds during the ScCO_2_ stage, likely due to the sustained temperature of 76 °C, consistent with de Andrade Lima et al. [35].

The strategic integration of ScCO_2_ and scW offers advantages beyond simple component recovery. Similar sequential approaches have been successfully applied to other matrices, such as the red calyxes of *Hibiscus sabdariffa* L. [36]. In that context, the initial ScCO_2_ phase serves as a structural pretreatment, effectively permeabilizing tegumental tissues, thereby mitigating internal mass transfer resistance for the subsequent diffusion of hydrophilic anthocyanins into the scW phase.

This “priming” effect—wherein the primary solvent conditions the biomass architecture for the secondary fluid—was reported to underpin the superior anthocyanin recovery observed in the *Hibiscus* study relative to conventional extraction benchmarks. These observations provide a compelling mechanical parallel to the significant efficiencies achieved in our pomegranate marc biorefining process, where the dual-solvent strategy facilitates the substantial recovery of both lipophilic and hydrophilic phytochemicals. Collectively, these results validate the reliability and scalability of the developed models, providing rigorous scientific scaffolding for the industrial valorisation of pomegranate processing residues into high-value nutraceutical ingredients.

### 2.4. Characterization of the Raw, Delipidated and Exhausted Pomegranate Matrices

The evolution of the proximate and bioactive composition across the sequential biorefining stages—from the raw feedstock to the final exhausted residue—is detailed in Table 11. The raw pomegranate marc was characterized by a baseline residual moisture of 10.4% and an ash content of 2.5%, serving as a complex matrix for several high-value components. The lipophilic fraction, totalling 44.8 ± 1.1 g kg^−1^, was accompanied by a robust profile of hydrophilic bioactives, including total phenolics [98.0 ± 8.8 g gallic acid equivalents (GAE) kg^−1^], soluble sugars (193.1 ± 1.3 g kg^−1^), functional polysaccharides (87.3 ± 2.1 g kg^−1^), and proteins (540 ± 10 mg kg^−1^). These constituents underpinned a significant antioxidant capacity of 623 ± 11 mmol Trolox equivalents (TE) kg^−1^. Within the lipid fraction, γ-tocopherol (80 ± 20 mg kg^−1^) was the sole detectable tocopherol isomer, while carotenoids remained below the limit of detection.

Following the ScCO_2_ extraction phase, the residual lipid content in the delipidated matrix was reduced to 15.2 ± 1.7 g kg^−1^. This reduction is quantitatively consistent with the 68% oleoresin extraction efficiency and corroborates the expected mass balance. The high selectivity of the supercritical fluid is evidenced by the preservation of non-lipid structural and bioactive components, as total phenolics (80.3 ± 7.0 g kg^−1^), soluble sugars (197.9 ± 2.3 g kg^−1^), polysaccharides (87.7 ± 2.1 g kg^−1^), and proteins (620 ± 60 mg kg^−1^) remained substantial. The antioxidant activity of the delipidated matrix increased to 679 ± 14 mmol TE kg^−1^, likely due to a relative enrichment effect resulting from lipid removal and the consequent concentration of polyphenols in the remaining dry matter.

The subsequent application of scW treatment yielded an exhausted matrix characterized by a near-total depletion of lipids. This exhaustive removal is likely the result of hydrothermal solubilization and minor lipid degradation occurring under subcritical conditions. The exhausted residue exhibited a marked decrease in hydrophilic solutes, with total phenolics, soluble sugars, and total polysaccharides declining to 42.3 ± 9.1 g kg^−1^, 62.5 ± 2.6 g kg^−1^, and 75.4 ± 2.0 g kg^−1^, respectively (*p* < 0.05). Consequently, the total antioxidant activity reached its minimum value of 328 ± 13 mmol TE kg^−1^. However, the relative retention of the polysaccharide fraction in the applied conditions suggests that while scW effectively recovered monomeric sugars, phenolics and part of polysaccharides, the lignocellulosic structural scaffold remained largely intact. This finding indicates that the exhausted marc, though depleted of primary bioactives, retains potential value as an insoluble fibre-rich byproduct or as a substrate for subsequent lignocellulosic conversion.

### 2.5. Comparative Characterization of Pomegranate Extracts: Conventional Sequential Solvent Extraction vs. Green ScCO_2_ and scW Biorefining

The detailed chemical profiling of the extracts obtained via sequential biorefining reveals a high degree of compositional fidelity and nutritional density, comparing favourably—and in some instances superiorly—to traditional solvent-based methodologies.

#### 2.5.1. Lipophilic Profiling: ScCO_2_ vs. Soxhlet Extraction

The fatty acid distribution of the oleoresin recovered through ScCO_2_ exhibited only minor deviations from the profiles obtained via conventional Soxhlet extraction using hexane, specifically limited to stearic, oleic, and linolenic acids (Table 12).

In both instances, the lipid fraction was dominated by unsaturated fatty acids (>91%), with conjugated linolenic acids (CLnAs) constituting over 72% of the total, consistent with the findings of Montefusco et al. [10]. Punicic acid (C_18:3_ *c*9, *t*11, *c*13) remained the primary constituent, followed by α-eleostearic, catalpic, β-eleostearic acid, and two unidentified CLnAs. Despite employing milder extraction conditions (40 MPa, 43 °C), Bustamante et al. [37] reported similar results, likely due to their use of 20% ethanol as a co-solvent, which enhances the polarity and extraction capacity of ScCO_2_, thereby facilitating higher yields even under less intensive operating conditions.

The health-promoting potential of these oleoresins was quantified using critical lipid quality indices. The PUFA/SFA ratios (7.94 for ScCO_2_ and 9.13 for Soxhlet) significantly exceed the minimum nutritional recommendation of 0.45 [38]. Furthermore, the low values for the Atherogenic Index (AI ≈ 0.08) and Thrombogenic Index (TI ≈ 0.60), coupled with a high hypo-/hypercholesterolemic ratio (h/H ≈ 14.25), highlight the oleoresin’s potential in mitigating cardiovascular risk factors [39,40]. Beyond the primary lipid profile, ScCO_2_ demonstrated a superior capacity for concentrating secondary bioactives (Table 13).

Specifically, γ-tocopherol levels were significantly higher in the ScCO_2_ extract (3.8 ± 0.9 g kg^−1^) compared to the hexane extract (2.44 ± 0.24 g kg^−1^), suggesting that the supercritical state may enhance the solubility and preservation of these thermolabile antioxidants, in agreement with Liu et al. [41].

#### 2.5.2. Hydrophilic Profiling: scW vs. Ethanolic Maceration

The scW extraction of the delipidated matrix yielded a dried extract with a significantly higher total phenolic concentration (160.4 ± 11.0 g GAE kg^−1^ dw) compared to 80% ethanolic maceration (Table 14). While the overall extraction efficiency from the raw matrix was higher for ethanol (50% vs. 69%), scW proved more effective at generating a highly concentrated final product. The scW extract exhibited a distinct hygroscopic, vitreous morphology, attributed to the co-extraction of soluble sugars (307.8 ± 41.9 g kg^−1^) and functional polysaccharides (8.3 ± 1.4 g kg^−1^), which were recovered at higher rates than in the ethanolic process.

**Figure 4:**
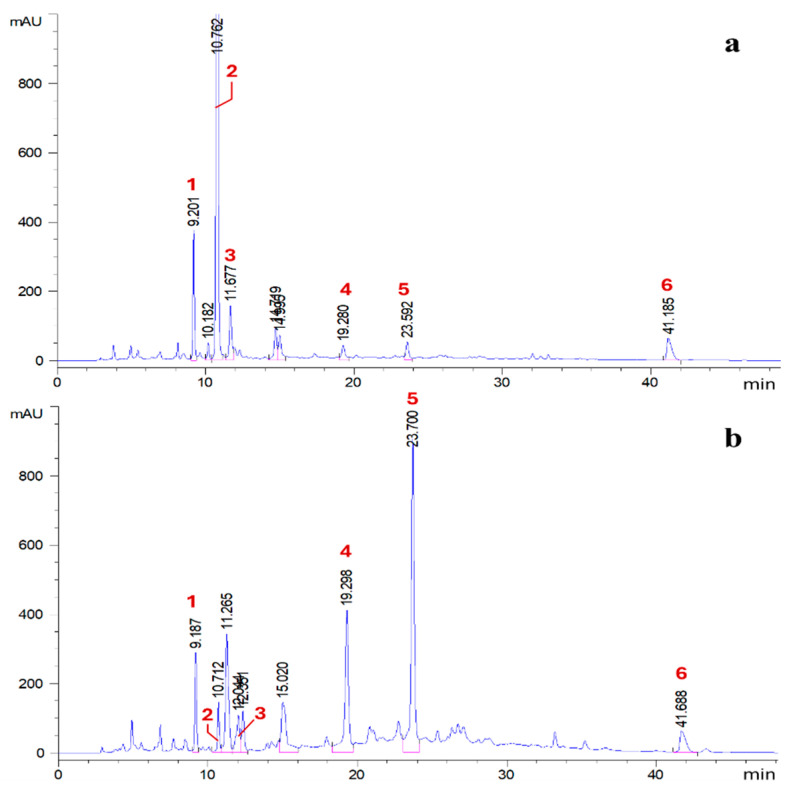
HPLC-DAD chromatograms of the phenolic profiles of the scW extract (**a**) and the 80% ethanol maceration extract (**b**). Identified peaks correspond to: 1—gallic acid, 2—glucogallin, 3—punicalin β, 4—punicalagin α, 5—punicalagin β, 6—ellagic acid. Peak identification was based on authentic standards, with retention times verified against literature data. Unlabelled peaks represent unidentified compounds.

The most striking divergence between the two methods was revealed by HPLC-DAD analysis (Figure 4 and Table 15).

The ethanolic maceration largely preserved the complex ellagitannin architecture, with punicalagin isomers (α and β) accounting for over 80% of the identified phenolics. Previous studies have shown that ethanol effectively preserves punicalagin isomers, supporting its suitability for recovering thermolabile phenolics [42]. In contrast, the scW profile was dominated by glucogallin (55.8%) and gallic acid, with a marked reduction in punicalagin content. This shift indicates that the hydrothermal conditions of scW (149 °C) promote the controlled hydrolysis of high-molecular-weight polyphenols into smaller derivatives.

This transition is driven by the unique physicochemical shifts in water in its subcritical state. At 149 °C, the significantly increased ionic product (*Kw*) of water promotes autoprotolysis, where the fluid acts as an intrinsic acid-base catalyst. This accelerates the hydrolysis of ester bonds in punicalagin, yielding smaller derivatives like gallic acid and glucogallin, whose potential bioavailability warrants further investigation [43]. Such mechanistic behaviour is consistent with previous reports describing tannin hydrolysis and phenolic degradation under subcritical water conditions [44].

Beyond this primary hydrolytic conversion, the hydrothermal conditions of scW may trigger secondary transformation pathways. One significant mechanism is the thermal decarboxylation of gallic acid, which can lead to the formation of pyrogallol, while the high pressure maintains the solubility of these increasingly less-polar intermediate metabolites. Additionally, at these temperatures, the oxidative cleavage of certain thermolabile flavonoids and the potential formation of Maillard reaction products (with sugars present in the extract) may occur.

While scW may induce the partial degradation of thermolabile compounds, it offers a unique “green” pathway for the controlled generation of specific bioactive metabolites without the use of organic solvents.

This susceptibility is aligned with their biological function, as many flavonoids in pomegranate peels are synthesized as reactive antioxidants and UV-protective pigments, making them inherently more prone to transformation under hydrothermal conditions. These pathways can differently affect antioxidant activity: the release of smaller phenolic units may enhance scavenging capacity, whereas the breakdown of complex tannins can reduce the synergistic interactions typical of the raw matrix. This suggests that the lower antioxidant capacity compared to ethanolic extraction reflects not only reduced phenolic yields but also these competing degradation and transformation mechanisms. Importantly, the preliminary ScCO_2_ treatment did not negatively impact subsequent phenolic recovery, with scW yields remaining statistically comparable between raw and delipidated matrices. This confirms the viability of the sequential ScCO_2_/scW framework as a sustainable, industrial-scale biorefining strategy for the exhaustive valorisation of pomegranate processing residues.

### 2.6. Comparative Analysis of Antioxidant Capacity: Conventional Solvent Maceration vs. Optimized Sequential ScCO_2_-scW Biorefining

To provide a rigorous benchmark for the integrated ScCO_2_/scW biorefining framework, a comparative performance analysis was conducted against a conventional, food-grade solvent extraction sequence. Ethyl acetate was utilized to recover lipophilic compounds due to its favourable safety profile in food applications, unlike hexane. For the hydrophilic stage, 80% ethanol was employed. The resulting extracts were further fractionated using solvents of varying polarity to map the distribution of antioxidant activity, as summarized in Table 16.

Quantitative yield data indicated that Soxhlet extraction with ethyl acetate achieved a higher total oleoresin recovery (42.3 ± 2.7 g kg^−1^) compared to the ScCO_2_ process (30.5 ± 2.4 g kg^−1^). This result is consistent with the exhaustive nature of classical reflux methods. However, the qualitative performance of these extracts varied significantly across fractions. Assessing antioxidant capacity through the TEAC (Trolox Equivalent Antioxidant Capacity) assay, the acetone fraction of the Soxhlet-derived oleoresin exhibited markedly higher activity than its ScCO_2_ counterpart. This disparity likely stems from the broader solvation power of boiling ethyl acetate, which may co-extract a wider range of moderately polar antioxidant compounds that are less soluble in pure ScCO_2_ under the investigated conditions.

In contrast, the highly non-polar hexane fractions for both methods demonstrated relatively low antioxidant activity in both TEAC and DPPH (2,2-diphenyl-1-picrylhydrazyl) assays. Interestingly, within this specific fraction, the ScCO_2_ extracts displayed slightly superior scavenging values compared to the Soxhlet-derived samples. This suggests that while ScCO_2_ may provide a lower total mass yield, its high selectivity potentially leads to a higher concentration of specific lipophilic antioxidants, such as tocopherols, which might undergo partial thermal degradation during the prolonged heating cycles characteristic of Soxhlet extraction.

A strong positive correlation was observed between γ-tocopherol levels and the radical scavenging activity of the hexane fraction (r = 0.80 for DPPH; r = 0.91 for TEAC) (Table S1). The correlation was even higher for total flavonoids, indicating that this phenolic subclass, together with tocopherols, is a primary driver of the observed bioactivity. This confirms that the antioxidant stability of the oleoresin is mechanistically driven by a synergistic effect within the lipophilic fraction; both tocopherols and flavonoids act as chain-breaking antioxidants, likely through a Hydrogen Atom Transfer (HAT) mechanism, effectively neutralizing peroxyl radicals.

Conversely, the negative correlations observed with the TEAC (Acetone) fraction (r = −0.69 for tocopherols and r = −0.88 for flavonoids) suggest that the polar environment of the acetone assay may favour different antioxidant species. The multivariate analysis further reveals a distinct separation between the structural fatty acid composition and the antioxidant capacity. The PUFA and CLnA (punicic acid) clusters, despite being the major constituents of the oleoresin, showed negligible correlation with radical scavenging activity (r ranging from 0.15 to 0.29). Thus, the high degree of unsaturation in the fatty acid backbone does not directly contribute to the extract’s primary antioxidant defense.

In contrast, MUFA and SFA exhibited strong positive correlations with the hexane-based antioxidant assays (r = 0.92 and 0.86 for DPPH, respectively). Mechanistically, this does not imply that saturated fats act as antioxidants; rather, it reflects that the extraction conditions (Soxhlet vs. ScCO_2_) that recover these lipid fractions also co-extract higher levels of γ tocopherol and flavonoids. These findings underscore the trade-off between total yield and extract refinement, reinforcing the role of ScCO_2_ as a targeted technology for producing high-purity, bioactive-rich fractions.

Maceration with 80% ethanol yielded a significantly higher total phenolic content (55.67 ± 2.82 g GAE kg^−1^ matrix) than scW extraction (40.22 ± 2.73 g GAE kg^−1^ matrix). This result confirms that ethanol extracts phenolics more efficiently under milder conditions. Furthermore, maceration-derived extracts displayed superior antioxidant activity across all assays, including TEAC in methanol, FRAP in water, and DPPH in ethanol. Pearson correlation analysis revealed that the antioxidant capacity of the PAE was governed by specific phenolic profiles rather than total yields. A robust positive correlation was observed between antioxidant outputs (TEAC, FRAP, DPPH) and punicalagins (α and β) as well as ellagic acid (r ≥ 0.86) (Table S1). Conversely, a strong negative correlation emerged with both total phenolic content (r ≤ −0.51) and glucogallin (r = −0.86). These findings suggest a mechanistic shift: while scW conditions facilitate the recovery of smaller hydrolysed units like gallic acid and glucogallin, these compounds exhibit lower radical scavenging efficiency compared to high-molecular-weight ellagitannins. The superior performance of ethanol maceration is attributed to the preservation of punicalagins, which utilize synergistic HAT and Single Electron Transfer (SET) pathways. In contrast, the thermal intensity of the scW process likely induces the degradation of these potent macro-molecules into less active monomeric units, thereby reducing the in vitro overall bioactive potential.

### 2.7. Conversion of Pomegranate Aqueous Extracts into Solid Dispersions via Spray Drying

**Figure 5:**
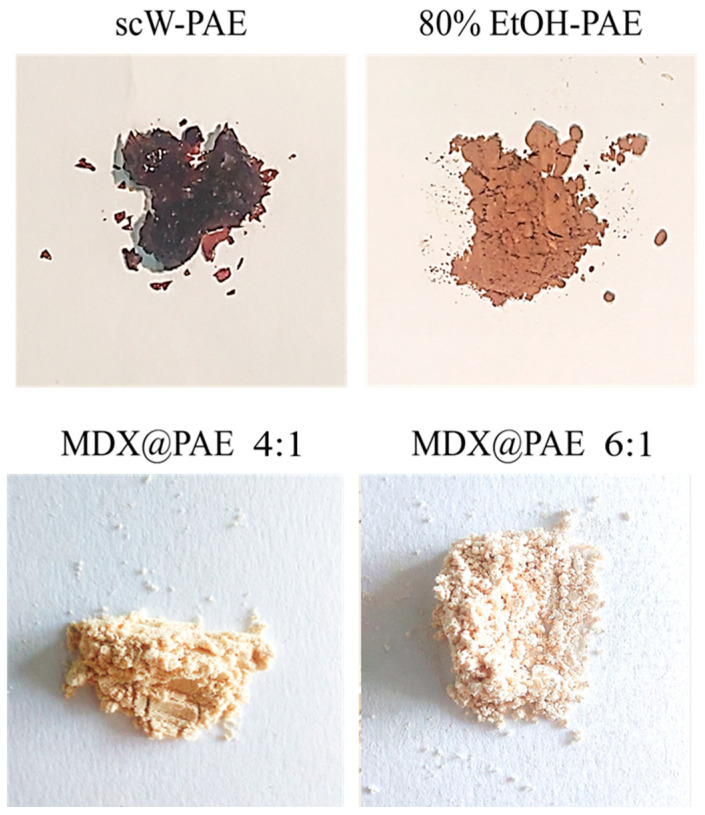
Macroscopic appearance of dried PAE obtained by scW (scW-PAE) and 80% ethanol maceration (80% EtOH-PAE), along with the solid dispersions produced by the incorporation of scW-PAE into a maltodextrin (MDX) matrix at carrier-to-phenolic ratios of 4:1 and 6:1 (MDX@PAE 4:1 and MDX@PAE 6:1, respectively).

Pomegranate aqueous extracts (PAE) derived from the sequential scW treatment of peels and seeds are characterized by a dense profile of phenolic compounds with documented antioxidant, anti-inflammatory, and antimicrobial potencies [45]. However, the physical transformation of the raw scW-PAE into a solid state presents significant industrial challenges. As illustrated in Figure 5, the transition from a liquid extract to a dry solid results in a dark brown, vitreous, and highly hygroscopic material. This stickiness is mainly caused by high concentration of low-molecular-weight sugars. As a result, the extract rapidly undergoes deliquescence and agglomeration, which compromises handling, storage stability, and downstream application.

To mitigate these rheological limitations, PAE was incorporated into a maltodextrin (MDX) matrix. Previous research has identified the core-to-carrier ratio as a critical parameter governing the physical state and recovery of solids during spray drying [46]. Preliminary trials showed that phenolic to maltodextrin ratios of 1:1 and 1:3 produced high stickiness and substantial wall deposition, preventing efficient powder recovery. Consequently, the formulation was adjusted toward higher carrier concentrations, evaluating two maltodextrin-to-phenolic ratios (4:1 and 6:1; MDX@PAE 4:1 and MDX@PAE 6:1). This approach transformed the sticky extract into a fine, free-flowing powder with improved handling.

The MDX@PAE 4:1 showed a phenolic retention efficiency (RE) of 98% and a production yield (PY) of 70%, whereas MDX@PAE 6:1 achieved 90% RE and a similar PY (71%). Residual moisture was 3.2 ± 0.4% (4:1) and 2.8 ± 0.3% (6:1), within the optimal stability range for spray-dried ingredients (<5%). These results indicate that the 4:1 ratio ensures near-quantitative recovery of the phenolic payload and is well-suited to the scW extract, enabling high retention without approaching carrier saturation.

**Figure 6:**
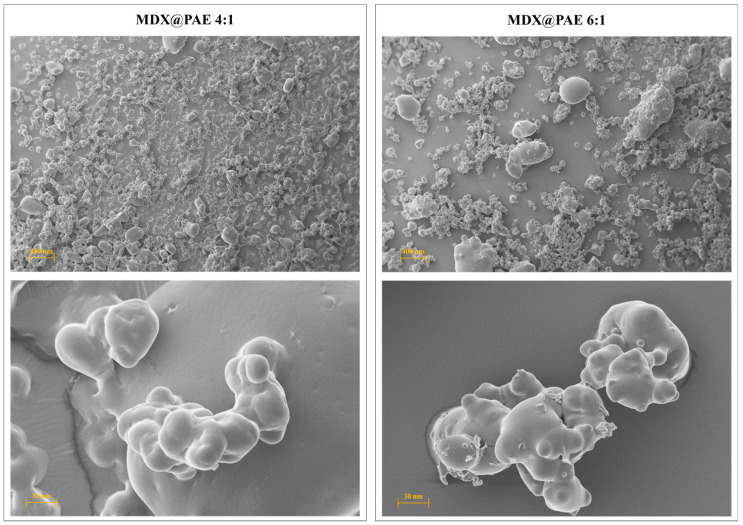
Scanning Electron Microscopy (SEM) images of PAE solid dispersion in maltodextrin (MDX@PAE) produced by spray drying. Micrographs depict particles at carrier-to-phenolic ratios of 6:1 and 4:1. Imaging conditions: EHT = 10.00 kV, WD = 9.65 mm, Probe Current = 100 pA. Scale bars represent 400 nm and 30 nm as indicated in the respective panels.

SEM (Figure 6) showed predominantly spherical, compact particles with smooth surfaces for both formulations. This morphology is typical of effective spray-drying and facilitates sequestration of the phytocomplex within the carbohydrate matrix. “While both ratios prevented significant structural defects such as surface cracking, the MDX@PAE 6:1 formulation exhibited a more heterogeneous size distribution, including smaller microstructures and occasional larger agglomerates. Conversely, the MDX@PAE 4:1 formulation produced a more uniform particle size distribution. This uniformity, combined with the higher phenolic retention, suggests that the 4:1 ratio is sufficient to form a cohesive solid dispersion while maintaining a higher density of bioactive compounds.

The application of maltodextrins as carriers is widely supported in the literature for converting liquid plant extracts into manageable solid forms, as they address common issues such as high solubility in water and poor flowability [47,48]. While maltodextrins are excellent for creating physical barriers and improving the handling of sensitive compounds, it should be noted that, as digestible polysaccharides, they may release the incorporated compounds in the upper gastrointestinal tract. Consequently, while these matrix-based systems are highly effective for standardization and stabilization of the extract’s physical state for nutraceutical or cosmetic ingredients, further development using composite materials could be explored for targeted or controlled-release applications.

### 2.8. Cytotoxic Assessment of Free PAE and Maltodextrin-Based Solid Dispersions on HEK-293 and MCF-7 Cell Lines

An exploratory screening of the cytotoxic potential of PAE obtained via scW—evaluated as a free extract and as maltodextrin-based solid dispersions (MDX@PAE)—was assessed to provide a preliminary toxicological profile using the MTT [*3*-(4,5-dimethylthiazol-2-yl)-2,5-diphenyltetrazolium bromide] colorimetric assay. Two human cell lines were utilized: Human Embryonic Kidney 293 (HEK-293), a model for immortalized but non-cancerous “normal” tissue, and Michigan Cancer Foundation-7 (MCF-7), an estrogen receptor-positive (ER+) breast adenocarcinoma model.

#### 2.8.1. Cytotoxicity of Free PAE

**Figure 7:**
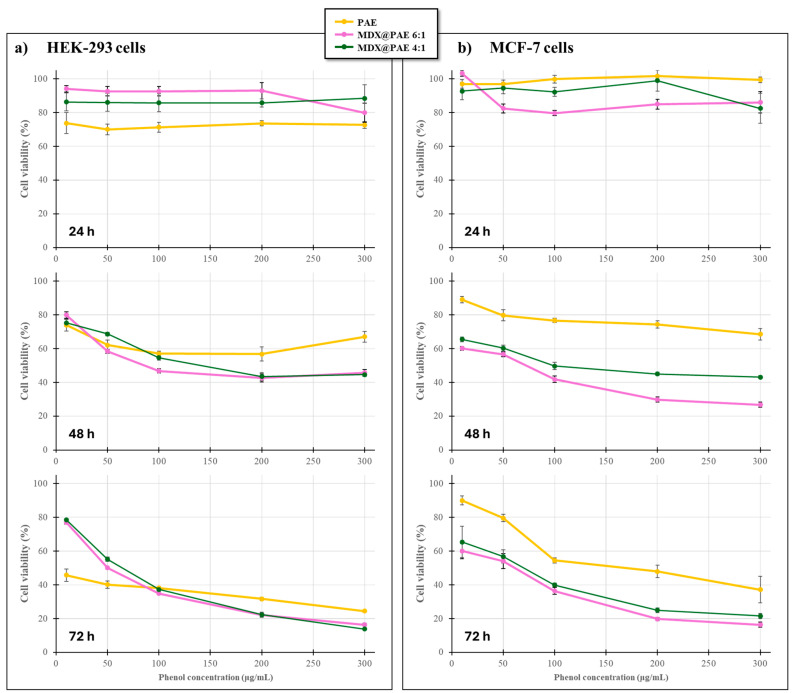
Cell viability (MTT assay) of HEK-293 (**a**) and MCF-7 (**b**) cells treated with free PAE or maltodextrin-based solid dispersions (MDX@PAE) at different ratios (6:1 and 4:1) after 24, 48 and 72 h. Data points represent the mean ± SD of three independent experiments (n = 3). Statistical significance was assessed by two-way ANOVA, followed by Holm–Sidak post hoc test for multiple comparisons to evaluate the effects of “Phenol concentration” and “Formulation type” on cell viability (see Table S2 for a summary). Differences were considered statistically significant at *p* < 0.05. Cell viability is expressed as a percentage of the optical density of the control group.

Exposure to free PAE resulted in time- and dose-dependent reductions in cell viability for both lineages (Figure 7). Notably, the non-cancerous HEK-293 line exhibited significantly higher sensitivity than the MCF-7 cancer line. After 24 h, MCF-7 cells maintained near-total viability, whereas HEK-293 viability decreased to approximately 75%. By 72 h, this trend intensified: MCF-7 viability remained above 50% for concentrations up to 200 μg mL^−1^, while HEK-293 viability plummeted to <40% at the maximum dosage (300 μg mL^−1^).

**Figure 8:**
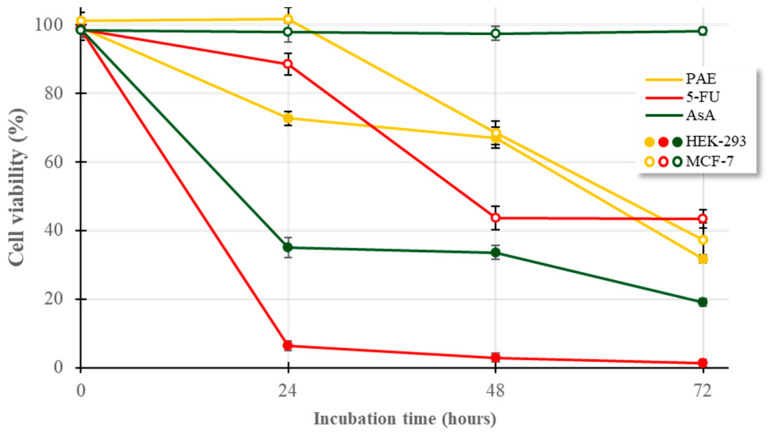
Cell viability (MTT assay) of HEK-293 and MCF-7 cells treated with 100 µM ascorbic acid (AsA) or 10 µM 5-fluorouracil (5-FU) over 72 h. Data points represent the mean ± SD of three independent experiments (n = 3). Statistical significance was assessed by two-way ANOVA, followed by the Holm–Sidak post hoc test for multiple comparisons to evaluate the effects of “Incubation time” and “Formulation type” on cell viability (see Table S3 for a summary). Differences were considered statistically significant at *p* < 0.05. Cell viability is expressed as a percentage of the optical density of the control group.

These results suggest that while PAE possesses bioactive properties, its intrinsic cytotoxicity is more pronounced in the rapidly proliferating, non-cancerous HEK-293 line. This differential sensitivity was further corroborated by the positive controls, 5-fluorouracil (5-FU) and ascorbic acid (AsA) (Figure 8).

#### 2.8.2. Response to Positive Controls (5-FU and AsA)

5-FU, a standard antimetabolite chemotherapeutic, induced rapid and severe cytotoxicity in HEK-293 cells, with >90% mortality within 24 h. Conversely, MCF-7 cells exhibited a more gradual decline, reaching 40% viability only after 48–72 h. This underscores a common clinical challenge: the lack of selectivity of 5-FU, which often results in significant off-target toxicity in healthy tissues. Similarly, AsA (a putative pro-oxidant at high concentrations [49]) reduced HEK-293 viability to <40% within 24 h, while MCF-7 cells remained largely unaffected (~90–100% viability). This resistance in MCF-7 may be attributed to overexpressed antioxidant defence systems, such as superoxide dismutase or glutathione peroxidase, which are characteristic of many malignant lineages [50]. Additionally, the high metabolic activity and deregulated cell cycle of immortalized HEK-293 cells may render them particularly vulnerable to these agents despite being non-cancerous [51].

#### 2.8.3. Influence of the Maltodextrin Matrix on Cytotoxic Activity

When MDX@PAE formulations were applied, the cytotoxic profiles were significantly modulated (Figure 7). These in vitro observations represent preliminary screening data and should not be interpreted as evidence of therapeutic potential. In HEK-293 cells, maltodextrin-based formulations at both 1:6 and 1:4 ratios exhibited reduced or comparable cytotoxicity relative to free PAE at lower concentrations (10–100 μg mL^−1^), suggesting a more favourable safety profile through controlled release or reduced immediate bioavailability. However, at higher concentrations (≥100 μg mL^−1^) and prolonged exposure (48–72 h), significant cytotoxicity persisted, indicating that the matrix formulation does not eliminate toxicity at elevated doses. Conversely, MDX@PAE formulations—particularly the 1:6 ratio—showed enhanced cytotoxicity in MCF-7 cells, consistently achieving the most pronounced reduction in cancer cell viability across all conditions.

These preliminary results suggest that formulating PAE as a solid dispersion may modify its cytotoxic profile, showing a concentration-dependent effect that partially reduces toxicity in non-cancerous cells only at lower doses. Nevertheless, the convergence of effects at higher doses indicates a need for further optimization. Future research should explore the molecular mechanisms underlying these observations—specifically regarding inflammation, oxidative stress, and apoptotic pathways—alongside in vivo studies to more comprehensively assess safety and functional efficacy [52].

## 3. Materials and Methods

### 3.1. Plant Material and Matrix Preparation

Fresh pomegranate marc, primarily consisting of peels and seeds (≈3:1 *w*/*w*), was obtained from the hydraulic pressing of *Punica granatum* fruits (var. Wonderful One). Multiple batches were provided by Cairo & Doutcher farm (Copertino, Lecce, Italy; coordinates: 40.298757, 18.022788) during the 2022–2023 harvest seasons.

**Figure 9:**
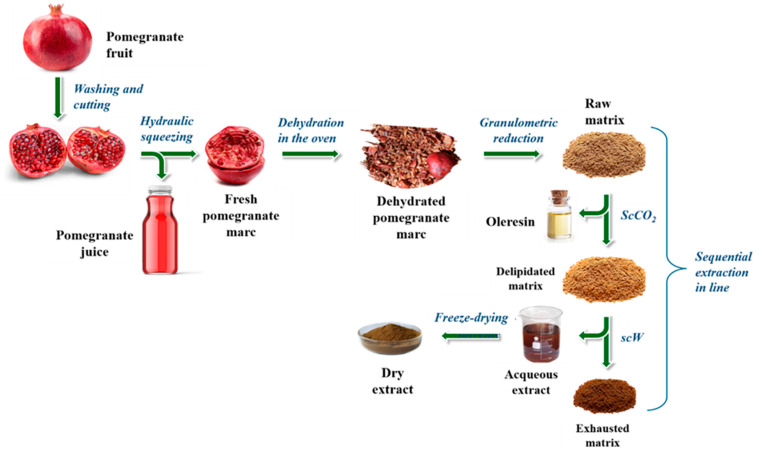
Process flow diagram illustrating the transformation of fresh pomegranate into the raw plant matrix, followed by the biorefining steps used to produce oleoresin and PAE.

The marc was dehydrated in a ventilated oven (Smeg, Guastalla, Reggio Emilia, Italy) at 60 °C for 48–72 h, then ground with a high-speed blender (Waring Laboratory Science, Torrington, CT, USA), and milled using an ultracentrifugal mill (ZM200, Retsch, Hann, Germany) equipped with a 35-mesh (500 µm) sieve (Figure 9). The resulting “raw pomegranate matrix” was vacuum-sealed in oxygen-impermeable, food-grade PA/PE plastic bags (Alpak, Taurisano, Lecce, Italy) and stored at −80 °C to ensure long-term stability.

### 3.2. Biochemical Characterization

Raw, delipidated, and exhausted matrices were characterized for moisture content, ash, lipids, isoprenoids, phenolics, flavonoids, carbohydrates, proteins, and antioxidant activity. All analyses were performed in triplicate. Statistical evaluation via one-way ANOVA confirmed no significant differences between batches (*p* > 0.05), allowing for the use of pooled averaged results. This approach allowed for the establishment of a robust, reproducible baseline for the sequential in-line process, minimizing the influence of raw material fluctuations on the optimization of extraction parameters.

#### 3.2.1. Determination of Residual Moisture and Ash Content

Moisture was determined by drying 1.0 g aliquots at 105 °C using a Büchi TO-50 dryer (Büchi Labortechnik AG, Flawil, Switzerland) until constant weight [53]. The moisture percentage was calculated as follows:

$$Moisture\;(\%)=\left(\frac{W_{initial}-W_{dry}}{W_{initial}}\right)\times 100$$

Ash content was measured gravimetrically by combustion at 550 °C for 3 h in a muffle furnace [54]. The ash content was expressed on a dry weight basis using the formula:

$$Ash\;(\%)=\left(\frac{W_{post-incineration}}{W_{initial}}\right)\times 100$$

#### 3.2.2. Determination of Total Lipid Content

The total lipid content of the pomegranate matrices was determined via Soxhlet extraction, following the protocol described by Shahidi [55]. Briefly, 50 g of each matrix was loaded into a cellulose thimble and subjected to exhaustive extraction using 275 mL of *n*-hexane for 8 h. Following extraction, the solvent was removed under reduced pressure using a rotary evaporator (R100, Büchi Labortechnik AG, Flawil, Switzerland) at 50 °C. The resulting oleoresins were dried to a constant weight and quantified using a high-precision analytical balance (PX224, Pioneer Analytical, OHAUS Europe GmbH, Nänikon, Switzerland). Total lipid content was expressed as g kg^−1^ matrix dw.

#### 3.2.3. Determination of Isoprenoid Content

Isoprenoids were determined following the method of Durante et al. [19]. Aliquots (0.1 g) of the oleoresins were dissolved in 1 mL of ethyl acetate and filtered through a 0.45 μm PTFE syringe filter (Millipore Corporation, Billerica, MA, USA).

Analyses were performed using an Agilent 1100 HPLC Series system (Agilent Technologies, Waldbronn, Germany) equipped with a reverse-phase C30 column (5 μm, 250 × 4.6 mm; YMC Inc., Wilmington, NC, USA). The mobile phase consisted of (A) methanol, (B) 0.2% ammonium acetate in methanol/water (80:20, *v*/*v*), and (C) methyl *tert*-butyl ether. The gradient elution was programmed as follows: 0 min: 95% A and 5% B; 0–12 min: 80% A, 5% B, and 15% C; 12–42 min: 30% A, 5% B, and 65% C; 42–60 min: 30% A, 5% B, and 65% C; 60–62 min: 95% A and 5% B. The column was re-equilibrated for 10 min between runs.

The flow rate was maintained at 1.0 mL min^−1^ at a column temperature of 25 °C, with an injection volume of 10 μL. Carotenoids and tocochromanols were monitored via a diode array detector (DAD) at 475 nm and 290 nm, respectively. Identification was performed by comparing retention times and UV–Vis spectra with authentic standards.

#### 3.2.4. Determination of Total Phenolic Content

Soluble phenolics were extracted according to Adom et al. [56] with minor modifications. Briefly, 0.5 g of matrix was homogenized with 10 mL of chilled 80% ethanol and stirred overnight at room temperature. The mixture was centrifuged at 3900× *g* for 10 min at 4 °C (Allegra™ X-22, Beckman Coulter, Inc., Brea, CA, USA). The pellet underwent a second extraction with 5 mL of 80% ethanol for 3 h. Supernatants were pooled for analysis.

Total phenolic content was quantified using the Folin–Ciocalteu method [57]. A 50 μL aliquot of the extract was mixed with 50 μL of Folin–Ciocalteu reagent and 450 μL of deionized water. After 5 min, 500 μL of 7% Na_2_CO_3_ and 150 μL of distilled water were added (final volume 1.25 mL). After 90 min of dark incubation, absorbance was measured at 750 nm (Beckman DU650 spectrophotometer, High Wycombe, UK). Results were expressed as gallic acid equivalents (GAE).

#### 3.2.5. Determination of Total Flavonoid Content

Total flavonoid content was determined using the aluminum chloride colorimetric assay [58]. Two grams of matrix were extracted with 7.5 mL of methanol in the dark (7 °C, 12–16 h). After centrifugation (4000× *g*, 10 min, 7 °C; Allegra™ X-22, Beckman Coulter), 50 µL of the supernatant was diluted with 450 µL of distilled water. Subsequently, 30 µL of 5% NaNO_2_ was added (5 min incubation), followed by 60 µL of 10% AlCl_3_ (6 min incubation). Finally, 200 µL of 1 M NaOH and 210 µL of distilled water were added. The mixture was vortexed, and absorbance was recorded at 510 nm. Results were expressed as catechin equivalents (CE).

#### 3.2.6. Determination of Total Soluble Sugar and Polysaccharide Content

Polysaccharide extraction followed the method of Zhu and Liu [59]. To remove soluble sugars, 0.4 g of matrix (raw, delipidated, or exhausted) was treated with 6 mL of 70% ethanol, shaken for 30 min at room temperature, and centrifuged at 2000× *g* for 10 min at 20 °C (Allegra™ X-22, Beckman Coulter, Inc., Brea, CA, USA). The supernatants from two successive washes were pooled for soluble sugar analysis.

The remaining pellets were washed three times with absolute ethanol, resuspended in 6 mL of distilled water (pH adjusted to 6.0–6.5), extracted at 100 °C for 90 min, and then centrifuged at 2000× *g* for 10 min at 20 °C. Both the water-soluble supernatant and the residual pellet (resuspended in 4 mL of distilled water) were retained for analysis to account for total polysaccharide content. Quantification was performed using the phenol-sulfuric acid method [60]. Briefly, 400 µL of each fraction was reacted with 10 µL of 80% phenol (*w*/*v*) and 1 mL of concentrated H_2_SO_4_. The mixture was incubated for a total of 20 min, with intermediate vortexing at 10 min and a final vortexing step. The absorbance was measured at 490 nm using a UV-2600 spectrophotometer (Shimadzu, Columbia, MD, USA). Results were calculated against a standard curve and expressed as g kg^−1^ dw.

#### 3.2.7. Protein Extraction and Quantification

Proteins were extracted using an enzymatic-assisted extraction [61]. One gram of matrix was incubated with 0.1 mL of Viscozyme^®^ L (>12 FBGU, Sigma-Aldrich, St. Louis, MO, USA) in 10 mL of water (pH 6.0) at 50 °C for 24 h. Subsequently, 20 mL of 0.5 M NaCl was added, the pH was adjusted to 9.25, and the slurry was stirred for 2 h. After centrifugation at 4500× *g* for 30 min at 4 °C (Allegra™ X-22, Beckman Coulter, Inc., Brea, CA, USA), the supernatant was acidified to pH 4.5 to precipitate proteins. The precipitate was collected via centrifugation, washed, adjusted to pH 7.2, and freeze-dried.

Protein concentration was determined by the Bradford microassay [62] using bovine serum albumin as a standard (5–50 µg mL^−1^). An 800 µL aliquot of the reconstituted isolate was mixed with 200 µL of Bradford reagent and incubated for 5 min before measuring absorbance at 595 nm. The contribution of Viscozyme^®^ L was determined separately and subtracted from the final values.

#### 3.2.8. Determination of Antioxidant Activity

The antioxidant capacity was determined via the Trolox Equivalent Antioxidant Capacity (TEAC) assay [63] using sequential extraction of hydrophilic and lipophilic fractions. Duplicate 0.4 g aliquots were extracted with 1 mL of methanol, shaken in the dark for 1 h, and centrifuged at 2900× *g* for 10 min at 10 °C (Heraeus Biofuge Primo, Thermo Fisher Scientific, Waltham, MA, USA). After collecting the methanolic (hydrophilic) supernatant, the pellet was sequentially re-extracted with 1 mL of acetone and 1 mL of *n*-hexane under identical conditions to obtain the pooled lipophilic fraction. The absorbance of both fractions was measured at 734 nm. Results were expressed as mmol Trolox equivalents (TE) kg^−1^.

### 3.3. Biochemical Profiling of Extracted Pomegranate Oleoresins

Oleoresins extracted via supercritical ScCO_2_ and conventional Soxhlet methods were characterized for their composition, including fatty acids, isoprenoids (carotenoids, tocopherols, and tocotrienols), total phenolics, flavonoids, soluble sugars, polysaccharides, and antioxidant activity. Analytical procedures were adapted from Section 3.2.4, Section 3.2.5, Section 3.2.6 and Section 3.2.8, with minor modifications as specified.

For total phenolic content, 0.2 g of oleoresin was extracted with 10 mL of 80% ethanol. Flavonoid content was determined by extracting 1 g of oleoresin with 4 mL of 100% methanol, followed by centrifugation at 3900× *g* for 10 min at 7 °C using an Allegra™ X-22 centrifuge (Beckman Coulter, Inc., Brea, CA, USA). Soluble sugars and polysaccharides were quantified from 0.1 g of oleoresin. Lipophilic antioxidant activity was assessed by dissolving 26 mg of oleoresin in 5 mL of acetone.

#### 3.3.1. Fatty Acid Analysis

Fatty acid profiles of oleoresins extracted via ScCO_2_ and Soxhlet were analyzed using GC–MS after derivatization to fatty acid methyl esters (FAMEs). For derivatization, 0.1 g of oleoresin was saponified with 3 mL of 0.5 M methanolic NaOH at 100 °C for 5 min. After cooling, 2 mL of 14% BF_3_ in methanol was added for methylation at 100 °C for 30 min. The reaction was cooled, followed by the addition of 1 mL of hexane and 1 mL of 0.6% NaCl solution. After centrifugation at 6000× *g* for 2 min at 4 °C, the upper organic phase containing FAMEs was collected. GC–MS analysis was performed using an Agilent 5977E system equipped with a DB-WAX capillary column (60 m × 0.25 mm i.d., 0.25 µm film thickness; Agilent Technologies, Santa Clara, CA, USA) as reported by Durante et al. [64].

#### 3.3.2. Calculation of Nutritional Indices

The nutritional quality of the lipid fraction was evaluated based on the polyunsaturated fatty acid/saturated fatty acid (PUFA/SFA) ratio and the calculation of three primary lipid indices: the atherogenicity index (AI), the thrombogenicity index (TI), and the hypo-/hypercholesterolemic (h/H) ratio, as described by Chen and Liu [65]. These indices were calculated using the following equations:

$$AI=\frac{C12:0+4\times C14:0+C16:0}{\sum UFA}$$

$$TI=\frac{C14:0+C16:0+C18:0}{\left(0.5\times MUFA\right)+\left(0.5\times n6PUFA\right)+\left(3\times n3PUFA\right)+(n3/n6)}$$

$$h/H=\frac{MUFA+PUFA}{C12:0+C14:0+C16:0}$$

where UFA represents unsaturated fatty acids, PUFA denotes polyunsaturated fatty acids, SFA is saturated fatty acids, MUFA refers to monounsaturated fatty acids.

### 3.4. Biochemical Profiling of Hydrophilic Extracts

Hydrophilic extracts, obtained via scW or maceration, were characterized for total phenolic content, flavonoid content, soluble sugars, polysaccharides, and antioxidant activity using the established protocols described above, with specific modifications. For total phenolic content, 200 µL of the extract was diluted with 800 µL of ethanol and centrifuged at 11,000× *g* for 5 min at 10 °C (Heraeus Biofuge Primo R, Thermo Fisher Scientific, Waltham, MA, USA). The resulting supernatant was analyzed as detailed in Section 3.2.4, using 50 µL of distilled water as a blank. For total flavonoid content, 7 mL of the extract was evaporated to dryness (R100, Büchi Labortechnik AG, Flawil, Switzerland), resuspended in 415 µL of methanol, and centrifuged at 3900× *g* for 10 min at 7 °C. The supernatant was analyzed as described in Section 3.2.5. Soluble sugars and polysaccharides were quantified from 0.1 g of dried extract following the procedures in Section 3.2.6. Hydrophilic antioxidant activity was evaluated following Section 3.2.8. Briefly, 200 µL of extract was diluted with methanol to reach 80% concentration, vortexed, and centrifuged at 3900× *g* for 5 min at 4 °C (Allegra™ X-22 centrifuge; Beckman Coulter, Inc., Brea, CA, USA). The supernatant was analyzed using the TEAC assay. The remaining pellet was resuspended in distilled water, vortexed, and analyzed. The antioxidant activities of the supernatant and pellet were combined to determine the total hydrophilic antioxidant activity.

### 3.5. Response Surface Analysis (RSM)

RSM was employed to optimize the extraction of lipophilic and hydrophilic phytocomplexes from pomegranate marc. This statistical approach allowed for the identification of optimal processing conditions while evaluating the significance of multiple independent variables and their interactions on the extraction yield [66]. A Box–Behnken Design (BBD) was implemented to explore the experimental space efficiently. The resulting data were fitted to polynomial regression models, and the statistical significance was evaluated via ANOVA at a confidence level of *p* < 0.05. Optimization was performed with the objective of maximizing extraction yields ($Y_{n}$) while minimizing independent variable ($X_{n}$) values where possible.

#### 3.5.1. RSM for ScCO_2_ Extraction

The ScCO_2_ extraction process was optimized using a BBD with four independent variables: pressure ($X_{1}$: 30–60 MPa), temperature ($X_{2}$: 40–80 °C), CO_2_ flow rate ($X_{3}$: 2–10 L min^−1^), and extraction time ($X_{4}$: 60–180 min). The ranges were selected based on preliminary trials and relevant literature [67], but also by carefully considering process-engineering constraints. These included equipment pressure limitations, CO_2_ density and solvent power variations under supercritical conditions, and mass-transfer kinetics—particularly the balance between solvent residence time and the intrinsic recalcitrance of the pomegranate marc matrix—to ensure operational stability, near-exhaustive recovery, and energy-efficient process performance.

A total of 30 trials were initially conducted, supplemented by 10 additional extractions in an augmented BBD to enhance model robustness, totalling 40 experimental runs (Table 17). To estimate the experimental variance, ten replicates of the center point (all variables at their intermediate levels) were performed. The statistical allocation of 6 degrees of freedom (d.f.) for ‘Pure Error’ reported in the ANOVA (Table 1) accounts for the model’s adjustment for block effects inherent in the augmented design, ensuring that the lack-of-fit and significance tests remain conservative and reliable. The response variable, $Y_{1}$, was defined as the oleoresin yield percentage relative to the total oleoresin content determined by exhaustive Soxhlet extraction (Section 3.2.2).

#### 3.5.2. RSM for scW Extraction

For scW extraction, a three-variable BBD was utilized, investigating the effects of temperature ($X_{1}$: 100–160 °C), water-to-solute ratio ($X_{2}$: 10–50 *v*/*w*), and extraction time ($X_{3}$: 25–120 min) based on prior established parameters [33], with the ranges further defined by process-engineering considerations, including the effect of temperature on water properties under subcritical conditions, mass-transfer limitations, and the need to avoid excessive degradation of the polysaccharide matrix, which could lead to reactor clogging, while ensuring efficient phenolic recovery.

The design comprised 15 initial trials and 4 additional replicates for robustness, totalling 19 runs (Table 18). Three replicates of the center point were performed to estimate the experimental error and ensure data reproducibility. The response variable, $Y_{2}$, represented the phenolic yield percentage relative to the total phenolic content of the post-CO_2_ matrix determined by exhaustive extraction with 80% ethanol.

#### 3.5.3. Data Encoding and Polynomial Model Fitting

To standardize the independent variables for both ScCO_2_ and scW models, data were encoded using the following transformation:

$$X=\frac{x-(x_{max}-x_{min})/2}{(x_{max}-x_{min})/2}$$

where x is the natural variable, X is the encoded variable, and x_max_ and x_min_ are the maximum and minimum values, respectively. The experimental data were fitted to the second-order polynomial equation:

$$Y=\beta_{0}+\sum \beta_{i}X_{i}+\sum \beta_{ii}X_{i}^{2}+\sum \beta_{ij}X_{i}X_{j}$$

where $\beta_{0}$ is the intercept, and $\beta_{i}$, $\beta_{ii}$, $\beta_{ij}$ represent the linear, quadratic, and interaction coefficients. Analysis was performed using Design-Expert^®^ software (v. 7.1.5, StatEase, Inc., Minneapolis, MN, USA) with a 95% confidence interval (*p* < 0.05).

### 3.6. Extraction System and Procedure

**Figure 10:**
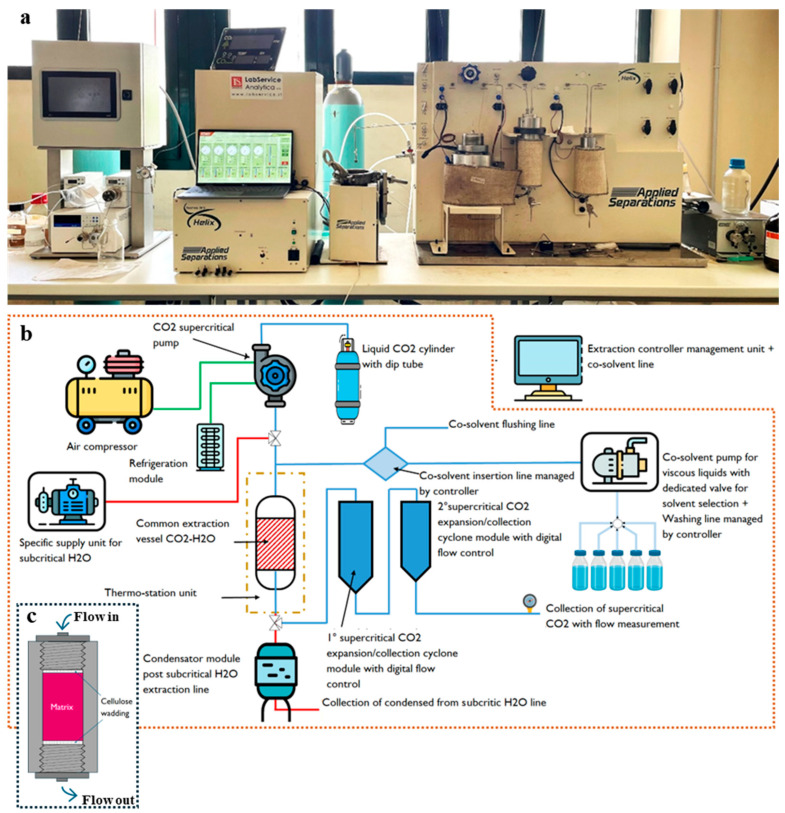
Photograph (**a**) and block diagram (**b**) of the combined ScCO_2_/scW Helix modular extraction system. Schematic representation of the extraction vessel loaded with the matrix and the inert (cellulose wadding). (**c**) Schematic representation of the extraction vessel loaded with the matrix and the inert (cellulose wadding).

The biorefining process utilized a modular Helix extraction system (Applied Separations, Allentown, PA, USA), customized for sequential ScCO_2_ and scW extractions in collaboration with LabService Analytica (Anzola dell’Emilia, Bologna, Italy; Figure 10).

#### 3.6.1. Lipophilic Extraction (ScCO_2_)

Pomegranate raw matrix (200 g) was loaded into a 500 cm^3^ stainless steel vessel ø = 7 cm; h = 25 cm). To prevent powdery matrix bypass, the vessel was padded with cellulose wadding cushions at both the inlet and outlet. The process consisted of a 30 min static phase for thermal equilibration, followed by a dynamic extraction phase at flow rates determined by the BBD. Oleoresins were collected in an expansion module at 20 MPa and 35 °C, weighed using a precision balance (AC 100, Mettler Toledo S.p.A., Milan, Italy), and the yield was calculated relative to Soxhlet extraction. Delipidated matrices were stored under vacuum in PA/PE bags (Alpak, Lecce, Italy) at −20 °C.

#### 3.6.2. Hydrophilic Extraction (scW)

The delipidated pomegranate matrices were pooled into a homogeneous batch for scW extraction. For each run, 50 g of matrix was loaded into the vessel under the same padding conditions. Extractions were conducted at a constant pressure of 4.0 MPa, following the temperature and time parameters specified in the BBD. The resulting PAE was collected via a condensation module, and aliquots were taken for volumetric measurement, dry weight determination, and total phenolic analysis.

### 3.7. Comparative Antioxidant Analysis

The innovative ScCO_2_/scW in-line process was compared against conventional sequential solvent extraction. The raw matrix was subjected to Soxhlet extraction using 100% ethyl acetate, followed by maceration with 80% ethanol. Antioxidant potential was characterized using FRAP and DPPH assays.

#### 3.7.1. Conventional Sequential Solvent Extraction

A 50 g sample of raw matrix was first extracted with 275 mL of ethyl acetate via Soxhlet. The oily extract was purified by decantation and concentrated (STRIKE 300, Steroglass S.r.l., Perugia, Italy). The remaining matrix was then extracted via maceration with 800 mL of 80% ethanol at 300 rpm for 24 h at room temperature. The mixture was centrifuged at 30,000× *g* for 15 min at 10 °C (JXN-26, Beckman Coulter, Inc., Brea, CA, USA), and the supernatant was concentrated to dryness.

#### 3.7.2. FRAP and DPPH Antioxidant Assays

Antioxidant activity was quantified using the Ferric Reducing Antioxidant Power (FRAP) assay [68] and the 2,2-diphenyl-1-picrylhydrazyl (DPPH) radical scavenging activity (DRSA) method [69]. For FRAP, 100 µL of sample was reacted with 3 mL of pre-warmed reagent (37 °C) and measured at 593 nm. For DPPH, a 60 µM ethanolic solution was reacted with the sample for 30 min in the dark. The radical scavenging percentage was calculated as:

$$\%DRSA=\left(1-\frac{A_{sample}}{A_{control}}\right)\times 100$$

where A_control_ is the absorbance of the DPPH solution without the sample.

### 3.8. Preparation of PAE Solid Dispersions via Spray-Drying

Optimized PAE was dehydrated using a Mini Spray Dryer S-300 (Büchi Labortechnik AG, Flawil, Switzerland), either as a pure extract or incorporated into maltodextrins (maltodextrin-to-phenolic ratios of 4:1 and 6:1). Spray-drying was performed using a standard two-fluid nozzle operated with an atomizing air flow of 1400 L/h and a drying gas flow of 25 m^3^/h. The inlet and outlet temperatures were controlled at 160 °C and 130 °C, respectively, while the feed solution was delivered at 12 mL/min, ensuring stable droplet formation and consistent drying efficiency.

Retention efficiency (RE) was determined to quantify the stability of the bioactive compounds during the dehydration process. This was calculated using the following equation:

$$RE\%=\left(\frac{{TPC}_{powder}\times {Yield}_{powder}}{{TPC}_{feed}}\right)$$

where ${TPC}_{powder}$ and ${TPC}_{feed}$ represent the total phenolic content in the final dehydrated product and the initial liquid feed, respectively. The dehydrated products were stored at −20 °C under vacuum in food-grade oxygen impermeable PA/PE plastic bags (Alpak, Taurisano, Lecce, Italy) until further use.

#### SEM Imaging of Spray-dried MDX@PAE Formulations

The morphology of the spray-dried MDX@PAE formulations was examined using an EVO 15 SEM (Carl Zeiss AG, Oberkochen, Germany). Samples were mounted on carbon adhesive stubs and gold-sputtered (40 nm) using a Balzers SCD 040 sputter coater (BAL-TEC AG, Balzers, Liechtenstein). Imaging was performed at an accelerating voltage of 10.00 kV under high vacuum.

### 3.9. In Vitro Cytotoxicity Evaluation of Free PAE and MDX-Based Solid Dispersions

The cytotoxic potential of free PAE and its maltodextrin-based formulations (MDX@PAE 1:6 and 1:4) was assessed using the MCF-7 (Michigan Cancer Foundation-7) and HEK-293 (Human Embryonic Kidney 293) cell lines.

Both cell lines were maintained in Dulbecco’s Modified Eagle Medium (DMEM), supplemented with 10% (*v*/*v*) Fetal Bovine Serum and 100 μg mL^−1^ penicillin-streptomycin. Cultures were incubated at 37 °C in a humidified atmosphere containing 5% CO_2_.

Cells were seeded into 96-well plates at specific densities optimized for their growth kinetics: 0.05 × 10^6^ cells mL^−1^ for HEK-293 and 0.10 × 10^6^ cells mL^−1^ for MCF-7 (100 μL per well). Following an overnight incubation to ensure surface adherence, the cells were exposed to varying concentrations of free PAE and MDX@PAE formulations (10, 50, 100, 200, and 300 μg mL^−1^, expressed as phenolic equivalents). Treatments were conducted over exposure intervals of 24, 48, and 72 h. Ascorbic acid (100 μM) and 5-fluorouracil (5-FU, 10 μM) were employed as positive controls, while ethanol-treated cells (matching the highest solvent concentration) served as an internal positive control.

Cell viability was quantified using the 3-(4,5-dimethylthiazol-2-yl)-2,5-diphenyltetrazolium bromide (MTT) colorimetric assay [69]. Briefly, 20 μL of MTT solution (final concentration: 0.5 mg mL^−1^) was added to each well and incubated at 37 °C in the dark for 3 h. During this period, metabolically active cells reduced the yellow tetrazolium salt into purple formazan crystals. Post-incubation, the supernatant was carefully aspirated, and the wells were rinsed with phosphate-buffered saline (PBS) to remove residual reagent. The formazan crystals were then solubilized in 100 μL of dimethyl sulfoxide (DMSO) under gentle agitation for 15 min at room temperature. Absorbance was recorded at 550 nm, with a background reference wavelength of 690 nm, using a multi-mode microplate reader. All experiments were performed in triplicate (n = 3), and cell viability was calculated relative to the untreated control group using the following equation:

$$Cell\;viability\;\left(\%\right)=\left(\frac{{Absorbance}_{treated\;cells}}{{Absorbance}_{control\;cells}}\right)\times 100$$

### 3.10. Statistical Analysis

Results are expressed as the mean value ± standard deviation of at least three independent experiments (n ≥ 3), as specified for each trial in the respective tables or figures. For the optimization of both ScCO_2_ and scW extraction processes, data were fitted to polynomial regression models using RSM, and the statistical significance of the models and their individual terms was evaluated via ANOVA at a confidence level of *p* < 0.05.

Significant differences between means for chemical characterization and antioxidant assays were established using Student’s t-test (for pairwise comparisons, e.g., ScCO_2_ vs. Soxhlet) or two-way ANOVA followed by the Holm–Sidak post hoc test for multiple comparisons. Pearson’s correlation analysis was also performed to identify mechanistic links between specific chemical constituents and antioxidant outputs. All statistical analyses and computational optimizations were performed using SigmaStat version 11.0 (Systat Software Inc., Chicago, IL, USA) and Design-Expert^®^ version 13 (Stat-Ease Inc., Minneapolis, MN, USA).

## 4. Conclusions

This study proposes a sustainable biorefinery framework for the integrated valorisation of pomegranate processing residues, evaluating the feasibility of an in-line sequential extraction strategy for recovering plant-derived compounds utilizing supercritical carbon dioxide (ScCO_2_) and subcritical water (scW). By leveraging the orthogonal solvating properties of these green fluids, the proposed methodology enables the differentiated recovery of high-value bioactives within a single, cohesive process. ScCO_2_ proved highly selective for the lipophilic fraction, yielding an oleoresin rich in punicic acid and γ-tocopherol, while the subsequent scW stage successfully addressed the hydrophilic payload of phenolics and functional carbohydrates.

A critical observation from this research is the inherent trade-off between extraction severity and chemical integrity. While the hydrothermal conditions of the subcritical water phase ensured significant recovery of solutes, the elevated temperatures induced a partial hydrolysis of complex ellagitannins into smaller phenolic derivatives, such as glucogallin. This transformation resulted in a moderate attenuation of total phenolic yields and antioxidant capacity relative to traditional ethanolic maceration. However, this shift in the phenolic profile suggests that scW could be strategically employed to generate specific metabolites with potentially distinct or enhanced biological activities, representing an underexplored avenue for the development of “designer” extracts.

To bridge the gap between raw extract recovery and industrial utility, the aqueous fractions were formulated as solid dispersions using a maltodextrin carrier via spray drying. This downstream intervention effectively mitigated the hygroscopic and vitreous nature of the scW extracts, transforming them into free-flowing powders. The results observed—particularly at the 4:1 maltodextrin-to-phenolic ratio—confirm that this matrix-based approach allows for the successful conversion of the extracts into a manageable solid form, significantly improving their handling properties and potential for inclusion in solid dosage systems.

The potential biological relevance of these formulations was explored through preliminary in vitro cytotoxicity assessments on HEK-293 and MCF-7 cell lines. These initial screenings suggested that the incorporation of PAE into a maltodextrin matrix might modulate the cellular response compared to the free extract, showing dosage-dependent variations in cell viability. While these results remain early-stage and exploratory, the matrix-based formulations (particularly the 6:1 ratio) appeared to show a trend toward reduced effects in the non-cancerous model at moderate doses, alongside a slight enhancement in cytotoxicity against the MCF-7 lineage. These observations provide a tentative indication that the physical state of the extract may influence its cellular interaction; however, given the sensitivity observed in the HEK-293 model, these findings are insufficient to draw definitive conclusions regarding the safety or toxicological profile of the extracts. Consequently, a comprehensive validation involving a broader panel of in vitro models and in vivo assessments remains necessary to establish safe exposure levels and confirm the suitability of these ingredients for commercial applications.

Looking toward industrial implementation, the transition from laboratory success to commercial scale requires further optimization of energy efficiency and the development of real-time monitoring systems to minimize thermal degradation. The industrial scalability of this sequential process is supported by high solvent recovery rates—typically exceeding 95% in closed-loop ScCO_2_ systems—and the elimination of energy-intensive organic solvent distillation. Specifically, the initial ScCO_2_ stage provides a ‘priming effect’ by increasing matrix porosity, which enhances mass transfer for the subsequent scW phase and optimizes overall energy consumption. Furthermore, while the current process proved robust for the standardized industrial batches investigated, significant fluctuations in raw material composition—arising from different cultivars, juice extraction methods, or seasonal shifts—would likely necessitate a recalibration of extraction parameters to ensure consistent efficiency. Nevertheless, this work provides a rigorous and environmentally benign scaffolding for the upcycling of agricultural by-products. By aligning green extraction technologies with advanced formulation strategies, this research advances the fundamental principles of the circular bioeconomy and offers a viable pathway to produce solvent-free, high-purity ingredients tailored for the nutraceutical, cosmetic, and pharmaceutical sectors.

## Figures and Tables

**Table 1:** Fit statistics for different predictive models and ANOVA for the quadratic response surface model regarding pomegranate oleoresin yield via ScCO_2_ extraction. The table compares linear, two-factor interaction (2FI), quadratic, and cubic models to identify the best fit for experimental data (bold). The ANOVA section details the significance of independent variables: pressure ($X_{1}$), temperature ($X_{2}$), CO_2_ flow rate ($X_{3}$), and extraction time ($X_{4}$), including their linear, quadratic and interaction terms. S.D., standard deviation; C.V.%, coefficient of variation; R^2^, coefficient of determination; R^2^ Adj., adjusted R^2^; R^2^ Pred., predicted R^2^; Adeq. Prec., adequate precision; d.f., degrees of freedom; Cor. Total, corrected total.

**Model**	**S.D.**	**C.V.%**	***p*** **-Value**	**Lack of Fit** ***p*** **-Value**	**R^2^**	**R^2^ Adj.**	**R^2^ Pred.**	**Adeq.** **Prec.**
Linear	0.4564	13.87	<0.0001	0.2337	0.5703	0.5166	0.3145	11.6480
2FI	0.4989	15.16	0.9912	0.1543	0.5829	0.4225	−0.0898	8.3502
**Quadratic**	**0.3631**	**11.04**	**0.0010**	**0.4994**	**0.8130**	**0.6940**	**0.3501**	**12.3051**
Cubic	0.3177	09.66	0.2939	0.7992	0.9479	0.7658		10.3607
**Source**	**Sum of squares**		**d.f.**	**Mean square**	***F*** **-value**	***p*** **-value**		
Block	4.07		3	1.36				
Quadratic model	12.61		14	0.9009	6.83	<0.0001	Significant	
X_1_—Pressure	0.3871		1	0.3871	2.94	0.1007		
X_2_—Temperature	3.99		1	3.99	30.23	<0.0001		
X_3_—CO_2_ flux	1.37		1	1.37	10.39	0.0039		
X_4_—Time	0.9912		1	0.9912	7.52	0.0119		
X_1_X_2_	0.0053		1	0.0053	0.0404	0.8426		
X_1_X_3_	0.0115		1	0.0115	0.0876	0.7700		
X_1_X_4_	0.2292		1	0.2292	1.74	0.2009		
X_2_X_3_	0.0207		1	0.0207	0.1570	0.6957		
X_2_X_4_	0.0001		1	0.0001	0.0007	0.9787		
X_3_X_4_	0.0197		1	0.0197	0.1496	0.7026		
X_1_^2^	0.1774		1	0.1774	1.35	0.2584		
X_2_^2^	1.16		1	1.16	8.80	0.0071		
X_3_^2^	1.63		1	1.63	12.38	0.0019		
X_4_^2^	0.6652		1	0.6652	5.05	0.0351		
Residual	2.90		22	0.1318				
Lack of Fit	2.15		16	0.1344	1.08	0.4994	not significant	
Pure Error	0.7494		6	0.1249				
Cor. Total	19.59		39					

**Table 2:** Fit statistics and ANOVA for the refined (reduced) quadratic model of pomegranate oleoresin yield. The model was simplified using an automatic forward selection procedure based on the *p*-value criterion (α = 0.10) to retain only the most impactful terms, thereby improving model parsimony and predictive power. The ANOVA results detail the significance of pressure (X_1_), temperature (X_2_), CO_2_ flux (X_3_), and extraction time (X_4_), alongside significant quadratic interactions. S.D., standard deviation; C.V.%, coefficient of variation; R^2^, coefficient of determination; R^2^ Adj., adjusted R^2^; R^2^ Pred., predicted R^2^; Adeq. Prec., adequate precision; d.f., degrees of freedom; Cor. Total, corrected total sum of squares.

Model	S.D.	C.V.%	R^2^	R^2^ Adj.	R^2^ Pred.	Adeq. Prec.
Modified quadratic	0.3392	10.31	0.7850	0.7331	0.5891	16.8211
Source	Sum of Squares	d.f.	Mean Square	*F* -Value	*p* -Value	
Block	4.07	3	1.36			
Model	12.18	7	1.74	15.12	<0.0001	Significant
X_1_—Pressure	0.7644	1	0.7644	6.64	0.0153	
X_2_—Temperature	5.13	1	5.13	44.58	<0.0001	
X_3_—CO_2_ flux	2.04	1	2.04	17.75	0.0002	
X_4_—Time	1.10	1	1.10	9.54	0.0044	
X_2_^2^	1.15	1	1.15	10.01	0.0036	
X_3_^2^	1.69	1	1.69	14.71	0.0006	
X_4_^2^	0.6977	1	0.6977	6.07	0.0200	
Residual	3.34	29	0.1150			
Lack of Fit	2.59	23	0.1125	0.9004	0.6134	Not significant
Pure Error	0.7494	6	0.1249			
Cor. Total	19.59	39				

**Table 3:** Goals, constraints and importance weights for optimization variables. Each variable has specified lower and upper limits with corresponding weights to emphasize their relative significance in the optimization process. $Y_{1}$ is a target response, while StdErr($Y_{1}$) indicates the acceptable range for standard error.

Name	Goal	Lower Limit	Upper Limit	Lower Weight	Upper Weight	Importance
X_1_—Pressure (Mpa)	Minimize	30	60	1	0.5	1
X_2_—Temperature (°C)	Minimize	40	80	1	0.5	1
X_3_—CO_2_ flux (L min^−1^)	Minimize	2	10	1	0.5	1
X_4_—Time (min)	Minimize	60	180	1	0.5	1
Y_1_—Yield % in oleoresin	Target	5.8	100.0	1	1	5
StdErr(Y_1_)	None	0.0918	0.1857	1	1	3

**Table 4:** Numerical optimization solutions for pomegranate oleoresin extraction parameters based on the desirability function. The table presents 17 potential configurations of pressure ($X_{1}$), temperature ($X_{2}$), CO_2_ flux ($X_{3}$), and extraction time ($X_{4}$) generated to maximize the oleoresin yield ($Y_{1}$). Solutions with the highest desirability score (Solutions 1–3) are highlighted in bold, representing the optimal balance between operational efficiency and extraction recovery. Sol. n., solution number; StdErr, standard error of the predicted mean; Des., desirability value (multi-objective optimization metric ranging from 0 to 1).

Sol. n.	X_1_—Pressure (Mpa)	X_2_—Temperature (°C)	X_3_—CO_2_ Flux (L min^−1^)	X_4_—Time (min)	Y_1_—Yield % in Oleoresin	StdErr(Y_1_)	Des.
**1**	**42.95**	**75.67**	**6.35**	**123.79**	**66.53**	**0.1109**	**0.706**
**2**	**43.07**	**75.66**	**6.36**	**123.85**	**66.64**	**0.1108**	**0.706**
**3**	**42.76**	**75.67**	**6.36**	**123.80**	**66.37**	**0.1110**	**0.706**
4	48.02	40.00	6.64	129.66	32.73	0.1394	0.637
5	48.11	40.00	6.65	129.75	32.78	0.1394	0.637
6	47.73	40.00	6.63	129.93	32.60	0.1393	0.637
7	47.78	40.00	6.67	129.46	32.64	0.1392	0.637
8	48.34	40.00	6.65	129.99	32.92	0.1396	0.637
9	48.17	40.00	6.69	129.84	32.89	0.1394	0.637
10	47.91	40.00	6.53	129.38	32.46	0.1395	0.637
11	48.40	40.00	6.64	131.81	33.12	0.1398	0.637
12	49.02	40.00	6.65	126.52	32.83	0.1398	0.637
13	46.43	40.00	6.56	129.36	31.84	0.1389	0.636
14	46.76	40.00	6.78	135.35	32.90	0.1390	0.636
15	46.52	40.00	6.99	128.03	32.32	0.1378	0.636
16	36.82	40.00	6.43	127.70	27.47	0.1465	0.625
17	34.96	40.00	6.81	129.91	27.51	0.1493	0.622

**Table 5:** Fit statistics for different predictive models and ANOVA for the linear response surface model regarding phenolic yield percentage from pomegranate marc. The table compares linear, two-factor interaction (2FI), quadratic, and cubic models, with the linear model (bold) identified as the most suitable fit for the experimental data. The ANOVA section evaluates the significance of subcritical water extraction parameters: temperature ($X_{1}$), water-to-solute ratio ($X_{2}$), and extraction time ($X_{3}$). S.D., standard deviation; C.V.%, coefficient of variation; R^2^, coefficient of determination; R^2^ Adj., adjusted R^2^; R^2^ Pred., predicted R^2^; Adeq. Prec., adequate precision; d.f., degrees of freedom; Cor. Total, corrected total sum of squares.

**Model**	**S.D.**	**C.V.%**	***p*** **-Value**	**Lack of Fit** ***p*** **-Value**	**R^2^**	**R^2^ Adj.**	**R^2^ Pred.**	**Adeq.** **Prec.**
**Linear**	**9.00**	**19.06**	**0.0002**	**0.3390**	**0.7154**	**0.6585**	**0.5004**	**11.5770**
2FI	9.54	20.21	0.7237	0.2691	0.7441	0.6161	0.0196	8.5986
Quadratic	8.25	17.48	0.1409	0.3805	0.8564	0.7127	−0.1002	9.5381
Cubic	7.45	15.77	0.3805		0.9480	0.7661		10.3173
**Source**	**Sum of squares**		**d.f.**	**Mean square**	***F*** **-value**	***p*** **-value**		
Linear model	3053.4		3	1017.71	12.57	0.0002	**Significant**	
X_1_—Temperature	1899.41		1	1899.41	23.46	0.0002		
X_2_—*v*/*w* Ratio	833.31		1	833.31	10.29	0.0059		
X_3_—Time	228.00		1	228.00	2.82	0.1140		
Residual	1214.39		15	80.96				
Lack of Fit	992.55		11	90.23	1.63	0.3390	not significant	
Pure Error	221.83		4	55.46				
Cor. Total	4267.53		18					

**Table 6:** Goals, constraints and importance weights for optimization variables. Each variable has specified lower and upper limits with corresponding weights to emphasize their relative significance in the optimization process. $Y_{2}$ is a target response, while StdErr ($Y_{2}$) indicates the acceptable range for standard error.

Name	Goal	Lower Limit	Upper Limit	Lower Weight	Upper Weight	Importance
X_1_—Temperature (°C)	Minimize	100	160	1	0.5	1
X_2_—*v*/*w* Ratio	Minimize	10	50	1	0.5	1
X_3_—Time (min)	Minimize	25	120	1	0.5	1
Y_2_—Yield % phenolics	Target	12.9	81.1	1	1	5
StdErr (Y_2_)	None	2.0955	5.0954	1	1	3

**Table 7:** Numerical optimization solutions for scW extraction of phenolic compounds from pomegranate marc. The table presents 2 potential configurations of temperature ($X_{1}$), water-to-solute ratio ($X_{2}$), and extraction time ($X_{3}$) generated to maximize the phenolic yield percentage ($Y_{2}$). Both solutions, which provide the highest desirability scores, are highlighted in bold, representing the optimal balance between operational efficiency and extraction recovery. Sol. n., solution number; StdErr, standard error of the predicted mean; Des., desirability value (multi-objective optimization metric ranging from 0 to 1).

Sol. n.	X_1_—Temperature (°C)	X_2_—Ratio Water/Solute (L kg^−1^)	X_3_—Time (min)	Y_2_—Yield % Phenolics	StdErr (Y_2_)	Des.
**1**	**149.30**	**39.60**	**72.72**	**58.56**	**2.83**	**0.615**
**2**	**149.29**	**39.60**	**73.08**	**58.60**	**2.83**	**0.615**

**Table 8:** Optimized operational parameters for the sequential biorefining of pomegranate marc using ScCO_2_ and scW.

Extraction	Pressure (MPa)	Temperature (°C)	CO_2_ Flux (L min^−1^)	Solvent/Solute Ratio (L kg^−1^)	Time (min)
**ScCO_2_**	43	76	6.4	-	124
**scW**	4	149	-	40	73

**Table 9:** Comprehensive mass balance of the sequential ScCO_2_/scW biorefining process for pomegranate marc. The table tracks the material distribution from the raw matrix input through lipophilic and hydrophilic extraction stages. The final fractions—oleoresin, dried hydrophilic extract, and exhausted residue—that constitute the total recovered mass are underlined. Data represent the mean ± standard deviation of at least three independent replicates (n ≥ 3).

Process Steps	Mass	
	Mass (g)	Mass (%)
Raw matrix input	200.0 ± 1.0	100
**ScCO_2_ extraction phase**		
Total oleoresin extracted	6.1 ± 0.5	3.0
Residual delipidated matrix	193.6 ± 0.8	96.8
**scW extraction phase**		
Total extracted material (dried)	48.8 ± 2.0	24.4
Dry exhausted matrix	144.8 ± 2.0	72.4
**Total final mass**	199.7 ± 4.0	99.8

**Table 10:** Validation of the optimized sequential extraction process: Comparison between experimental and model-predicted yields for oleoresin and total phenolic content. Experimental values represent the results obtained under the optimal conditions identified by RSM for both ScCO_2_ and scW stages. Data are presented as mean ± standard deviation of at least three independent determinations (n ≥ 3).

Target Fraction	Total Content in Matrix (g kg^−1^)	Total Extracted Material (g kg^−1^ dw)	Experimental Yield (%)	Model Predicted Yield (%)
**Oleoresin (ScCO_2_)**	44.8 ± 1.1	30.5 ± 2.4	68.1	68
**Total phenolics (scW)**	80.3 ± 8.8	47.1 ± 6.9	58.7	59

**Table 11:** Biochemical characterization of pomegranate marc across the sequential biorefining stages. The table compares the raw plant matrix, the delipidated matrix (following optimized ScCO_2_ extraction), and the exhausted matrix (following optimized scW extraction). Data are expressed as mean ± standard deviation (n ≥ 3). Different superscript letters within a row indicate statistically significant differences among matrices according to one-way ANOVA followed by the Holm–Sidak post hoc test (*p* < 0.05). GAE, gallic acid equivalents; TE, Trolox equivalents; n.d., not detected.

	Matrix		
	Raw	Delipidated	Exhausted
**Residual moisture (%)**	10.4 ± 0.2 ^a^	10.1 ± 0.1 ^a^	5.48 ± 1.4 ^b^
**Ash (%)**	2.5 ± 0.1 ^a^	1.2 ± 0.4 ^b^	1.4 ± 0.5 ^b^
**Total lipids (g kg^−1^)**	44.8 ± 1.1 ^a^	15.2 ± 1.7 ^b^	n.d.
**γ-Tocopherol (mg kg^−1^)**	80 ± 20	n.d.	n.d.
**Total phenolics (g GAE kg^−1^)**	98.0 ± 8.8 ^a^	80.3 ± 7.0 ^b^	42.3 ± 9.1 ^c^
**Total carbohydrates (g kg^−1^)**	280.4 ± 4.4 ^a^	285.6 ± 5.4 ^a^	137.9 ± 4.6 ^b^
** ** **Total soluble sugars (g kg^−1^)**	193.1 ± 2.3 ^a^	197.9 ± 3.3 ^a^	62.5 ± 2.6 ^b^
** ** **Total polysaccharides (g kg^−1^)**	87.3 ± 2.1 ^a^	87.7 ± 2.1 ^a^	75.4 ± 2.0 ^b^
**Proteins (mg kg^−1^)**	540 ± 10 ^a^	620 ± 60 ^ab^	720 ± 85 ^b^
**Total antioxidant activity (mmol TE kg^−1^)**	623 ± 11 ^b^	679 ± 14 ^a^	328 ± 13 ^c^

**Table 12:** Fatty acid profile and lipid quality indices of pomegranate oleoresin extracted via Soxhlet (hexane) and optimized ScCO_2_. Results are expressed as a percentage of total fatty acids (mean ± standard deviation, n ≥ 3). Different superscript letters within the same row indicate statistically significant differences between extraction methods (Student’s *t*-test, *p* < 0.05). SFA, saturated fatty acids; MUFA, monounsaturated fatty acids; PUFA, polyunsaturated fatty acids; CLnA, conjugated linolenic acids; AI, atherogenic index; TI, thrombogenic index; h/H, hypo-/hyper-cholesterolemic ratio. Dimensionless ratios are presented in italics.

	Oleoresin	
Saturated Fatty Acids (SFA)	Soxhlet	ScCO_2_
Myristic (C14:0)	0.21 ± 0.04 ^a^	0.15 ± 0.02 ^a^
Pentadecanoic (C15:0)	0.07 ± 0.02 ^a^	0.05 ± 0.00 ^a^
Palmitic (C16:0)	5.63 ± 0.50 ^a^	6.09 ± 0.40 ^a^
Heptadecanoic (C17:0)	0.11 ± 0.02 ^a^	0.15 ± 0.05 ^a^
Stearic (C18:0)	2.32 ± 0.08 ^b^	2.98 ± 0.15 ^a^
Arachidic (C20:0)	0.74 ± 0.09 ^a^	0.65 ± 0.05 ^a^
**Total SFA**	**9.08 ± 0.75** ^a^	**10.07 ± 0.67** ^a^
**Mono-unsaturated fatty acids (MUFA)**		
Palmitoleic (C16:1 n-7)	0.09 ± 0.01 ^a^	0.07 ± 0.01 ^a^
*cis*-10-Heptadecenoic (C17:1 n-7c)	0.07 ± 0.01 ^a^	0.12 ± 0.05 ^a^
Oleic (C18:1 n-9c)	5.56 ± 0.30 ^b^	6.63 ± 0.10 ^a^
*cis*-11-Octadecenoic (C18:1 n-7c)	0.69 ± 0.06 ^a^	0.72 ± 0.02 ^a^
*cis*-13-Eicosenoic (C20:1 n-7c)	0.69 ± 0.21 ^a^	0.58 ± 0.20 ^a^
*cis*-11-Eicosenoic (C20:1 n-9)	0.84 ± 0.08 ^a^	0.82 ± 0.07 ^a^
**Total MUFA**	**7.94 ± 0.67** ^a^	**8.94 ± 0.45** ^a^
**Polyunsaturated fatty acids (PUFA)**		
Linoleic (C18:2 n-6)	9.83 ± 0.89 ^a^	9.50 ± 0.60 ^a^
Linolenic (C18:3 n-3)	1.05 ± 0.21 ^a^	0.37 ± 0.04 ^b^
Punicic (C18:3 n-5, 9c, 11t, 13c)	32.04 ± 2.03 ^a^	29.23 ± 4.6 ^a^
α-Eleostearic (C18:3 n-5, 9c, 11t, 13t)	13.73 ± 0.53 ^a^	12.73 ± 0.4 ^a^
Catalpic (C18:3 n-5, 9t, 11t, 13c)	10.71 ± 0.73 ^a^	12.92 ± 3.5 ^a^
β-Eleostearic (C18:3 n-5, 9t, 11t, 13t)	8.09 ± 0.69 ^a^	7.77 ± 0.35 ^a^
CLnA not identified 1	4.38 ± 0.22 ^a^	4.46 ± 0.25 ^a^
CLnA not identified 2	3.09 ± 0.19 ^a^	3.00 ± 0.17 ^a^
**Total PUFA**	**82.92 ± 5.49** ^a^	**79.98 ± 9.91** ^a^
**Total CLnA**	**72.04 ± 4.39** ^a^	**70.11 ± 9.27** ^a^
**Lipidic quality indices**		
*PUFA/SFA*	*9.13 ± 0.97* ^a^	*7.94 ± 1.51* ^a^
*AI*	*0.07 ± 0.01* ^a^	*0.08 ± 0.01* ^a^
*TI*	*0.48 ± 0.07* ^a^	*0.60 ± 0.08* ^a^
*h/H*	*15.56 ± 1.78* ^a^	*14.25 ± 2.62* ^a^

**Table 13:** Comparison of γ-tocopherol, flavonoid, and carbohydrate content in pomegranate oleoresins obtained via optimized ScCO_2_ and Soxhlet (hexane) extraction. The table presents the concentration of bioactives relative to both the oleoresin mass and the starting raw matrix, along with the percentage yield relative to the total available content in the plant material. Values are expressed as mean ± standard deviation (n ≥ 3). Statistically significant differences between the two methods (Student’s *t*-test, *p* < 0.05) are indicated by an asterisk (*).

	ScCO_2_ (Optimized)			Soxhlet (Hexane)		
	g kg^−1^ Oleo.	g kg^−1^ Matrix	Yield %	g kg^−1^ Oleo.	g kg^−1^ Matrix	Yield %
**γ-Tocopherol**	3.8 ± 0.9 *	0.08 ± 0.02	100%	2.4 ± 0.2	0.07 ± 0.01	87.5%
**Total flavonoids**	34.2 ± 2.2 *	0.75 ± 0.05	4.12%	24.2 ± 1.7	0.73 ± 0.05	4.01%
**Total carbohydrates**	11.6 ± 2.6	0.25 ± 0.06	0.09%	11.1 ± 0.4	0.63 ± 0.02	0.22%
Soluble sugars	7.0 ± 1.3	0.15 ± 0.03	0.08%	6.7 ± 0.1	0.38 ± 0.00	0.19%
Polysaccharides	4.6 ± 1.3	0.10 ± 0.03	0.11%	4.4 ± 0.3	0.25 ± 0.02	0.29%

**Table 14:** Comparison of bioactive and carbohydrate profiles between scW extracts and traditional ethanol maceration (80% *v*/*v*) of pomegranate marc. The table evaluates the extraction efficiency relative to the extract mass and the original raw matrix, including the recovery yield percentage for total phenolics, flavonoids, and carbohydrates. Results are expressed as mean ± standard deviation (n ≥ 3). Statistically significant differences between the two methods (Student’s *t*-test, *p* < 0.05) are indicated by an asterisk (*).

	80% Ethanol Maceration			scW		
	g kg^−1^ Extract	g kg^−1^ Matrix	Yield %	g kg^−1^ Extract	g kg^−1^ Matrix	Yield %
**Total phenolics**	140.3 ± 7.1	55.7 ± 2.8 *	69%	160.4 ± 11 *	40.2 ± 2.7	50%
**Total flavonoids**	47.8 ± 5.7	19.0 ± 2.3	97%	70.8 ± 1.5 *	17.8 ± 0.3	91%
**Total carbohydrates**	245.0 ± 21.5	97.2 ± 8.5	34%	316.0 ± 43.4	79.6 ± 10.9	28%
Soluble sugars	242.0 ± 20.6	96.0 ± 8.2	49%	307.8 ± 41.9	77.5 ± 10.6	39%
Polysaccharides	2.97 ± 0.86	1.2 ± 0.3	1.3%	8.3 ± 1.4 *	2.1 ± 0.4 *	2.2%

**Table 15:** Individual phenolic profile of pomegranate marc extracts obtained via scW extraction and ethanol maceration (80% *v*/*v*). Quantification of specific hydrolysable tannins and phenolic acids was performed using HPLC-DAD. Values are expressed as mg per gram of starting matrix and as a percentage of the total identified phenolic fraction. Data represent mean ± standard deviation (n ≥ 3). Different superscript letters within a row indicate statistically significant differences between extraction methods (Holm–Sidak post hoc test, *p* < 0.05).

Compound	Maceration (80% EtOH)		scW	
	mg g^−1^ Matrix	%	mg g^−1^ Matrix	%
Punicalagin α	25.1 ± 3.4 ^a^	38.2	8.1 ± 0.9 ^b^	17.3
Punicalagin β	28 ± 4.2 ^a^	42.7	1.7 ± 0.5 ^b^	3.6
Punicalin β	4.1 ± 0.8 ^b^	6.2	6.3 ± 0.3 ^a^	13.3
Gallic acid	1.4 ± 0.3 ^b^	2.2	2.1 ± 0.2 ^a^	4.5
Glucogallin	3.6 ± 0.6 ^b^	5.5	26.3 ± 4.7 ^a^	55.8
Ellagic acid	3.4 ± 0.6 ^a^	5.2	2.6 ± 0.3 ^a^	5.5
**Total identified**	65.6 ± 9.9 ^a^		47.1 ± 6.9 ^b^	

**Table 16:** Antioxidant capacity of lipophilic and hydrophilic fractions obtained from pomegranate marc via sequential green extraction (ScCO_2_/scW) and conventional methods. (**a**) Lipophilic extracts: comparison between optimized ScCO_2_ and Soxhlet (ethyl acetate) extraction. (**b**) Hydrophilic extracts: comparison between optimized scW extraction and 80% ethanol maceration. Antioxidant activity is assessed via TEAC, DPPH, and FRAP assays. Values are mean ± standard deviation (n ≥ 3). Different superscript letters within the same row (for each sub-table) indicate statistically significant differences (*p* < 0.05). GAE, gallic acid equivalents; TE, Trolox equivalents.

**(a)** **Extraction Method** **(Lipophilic Compounds)**	**Total Oleoresin** **(g kg^−1^ Matrix)**	**Antioxidant Activity (mmol TE kg^−1^ Oleoresin)**		
		**TEAC** **(Acetone)**	**TEAC** **(Hexane)**	**DPPH** **(Hexane)**
Soxhlet (EtOAc)	42.3 ± 2.7	82.6 ± 6.1 ^a^	1.09 ± 0.03 ^b^	1.90 ± 0.07 ^b^
ScCO_2_	30.5 ± 2.4 ^b^	24.1 ± 2.1 ^b^	1.28 ± 0.04 ^a^	2.08 ± 0.02 ^a^
**(b)** **Extraction method** **(hydrophilic compounds)**	**Total phenolics** **(g GAE kg^−1^ matrix)**	**Antioxidant activity (mmol TE kg^−1^ matrix)**		
		**TEAC** **(Methanol)**	**FRAP** **(Water)**	**DPPH** **(Ethanol)**
Maceration (80% EtOH)	55.67 ± 2.82 ^a^	1169 ± 92 ^a^	1026 ± 77 ^a^	551 ± 79 ^a^
scW	40.22 ± 2.73 ^b^	741 ± 45 ^b^	225 ± 42 ^b^	95 ± 27 ^b^

**Table 17:** (BBD) matrix for the optimization of lipophilic oleoresin extraction from pomegranate marc using ScCO_2_. The table displays the experimental runs involving four independent variables: Pressure ($X_{1}$), Temperature ($X_{2}$), CO_2_ flow rate ($X_{3}$), and Extraction time ($X_{4}$). $Y_{1}$ represents the oleoresin yield percentage, comparing observed experimental values with those predicted by the quadratic response surface model.

Run n.	Independent Variables				Y_1_ Yield % in Oleoresin	
	X_1_—Pressure	X_2_—Temperature	X_3_—CO_2_ Flow Rate	X_4_—Time		
	MPa	°C	L min^−1^	min	Observed	Predicted
1	60	40	6	120	28.9	18.4
2	45	60	2	180	11.8	8.3
3	60	80	6	120	67.9	54.1
4	30	80	6	120	25.3	35.5
5	45	60	6	120	12.2	17.8
6	30	40	6	120	7.7	12.2
7	45	60	10	60	8.1	9.2
8	45	60	10	180	16.8	16.4
9	45	60	2	60	5.8	4.7
10	45	60	6	120	18.2	17.8
11	45	40	2	120	15.4	14.9
12	45	60	6	120	22.2	38.5
13	45	80	10	120	80	85.6
14	45	60	6	120	56.8	38.5
15	45	80	2	120	51.9	43.4
16	45	40	10	120	36.8	29.4
17	60	60	6	180	27.8	47.9
18	60	60	6	60	35.7	26.8
19	30	60	6	180	33.2	31.5
20	30	60	6	60	17.6	17.6
21	30	60	2	120	22.2	14.0
22	60	60	10	120	34.1	41.7
23	45	40	6	180	31.6	31.5
24	60	60	2	120	10.4	21.3
25	45	40	6	60	14.2	17.6
26	45	80	6	180	104.4	92.8
27	45	60	6	120	48.6	37.3
28	30	60	10	120	31.4	27.7
29	45	60	6	120	30.9	37.3
30	45	80	6	60	74.6	51.9
31	45	60	6	120	38.6	43.8
32	30	80	2	60	14.2	22.6
33	60	80	10	60	67.2	68.0
34	45	60	6	120	57.2	43.8
35	60	40	10	60	22.9	23.1
36	45	60	6	120	46.4	43.8
37	60	80	2	60	34.4	34.5
38	30	40	10	60	15.8	15.3
39	45	60	6	120	60.3	43.8
40	60	40	2	60	11.1	11.8

**Table 18:** BBD matrix for the optimization of scW extraction of phenolic compounds from pomegranate marc. The table displays the experimental runs for three independent variables: Temperature ($X_{1}$), Water/Solute ratio ($X_{2}$), and Extraction time ($X_{3}$). $Y_{2}$ represents the phenolic yield percentage, comparing the values observed experimentally with those predicted by the quadratic response surface model.

Run n.	Independent Variables			Y_2_ Yield % Phenolics	
	X_1_—Temperature	X_2_—Ratio Water/Solute	X_3_—Time		
	°C	L Kg^−1^	Min	Observed	Predicted
1	100	10	72.5	29.4	24.5
2	160	10	25	47.2	45.5
3	100	30	120	30.1	37.9
4	100	10	120	31.1	29.1
5	100	30	25	12.9	28.7
6	160	10	120	41	54.7
7	100	50	72.5	52.1	42.1
8	160	30	72.5	63.6	58.9
9	160	50	25	52.4	63.1
10	160	30	72.5	53.6	58.9
11	160	30	72.5	66.7	58.9
12	160	50	120	81.1	72.3
13	130	30	72.5	59.6	46.1
14	130	30	72.5	45.7	46.1
15	130	10	25	38.7	32.7
16	130	30	72.5	45.8	46.1
17	130	50	25	51.5	50.3
18	130	50	120	51.8	59.5
19	130	10	120	42.7	41.9

## Data Availability

The original contributions presented in this study are included in the article/Supplementary Material. Further inquiries can be directed to the corresponding author.

## Supplementary Materials

The following supporting information can be downloaded at: https://www.mdpi.com/article/10.3390/plants15060951/s1, Table S1. Pearson correlation coefficients (R) linking major bioactive markers to antioxidant capacity. Bold values indicate significant correlations (r > 0.80; *p* < 0.05); Table S2. Two-way ANOVA summary for Figure 7: Statistical significance was assessed by Two-way ANOVA, followed by the Holm–Sidak post-hoc test for multiple comparisons (n = 3). Factors evaluated: “Phenol concentration” and “Formulation type” for dose-response data. The threshold for statistical significance was set at *p* < 0.05; Table S3. Two-way ANOVA summary for Figure 7: Statistical significance was assessed by Two-way ANOVA, followed by the Holm–Sidak post-hoc test for multiple comparisons (n = 3). Factors evaluated: “Incubation time” and “Treatment type” for time-course data (Figure 8). The threshold for statistical significance was set at *p* < 0.05.
