# Supplementary material for: Sequential Supercritical CO2 and Subcritical Water Extraction for the Valorisation of Pomegranate (Punica granatum L.) By-Products: A Response Surface Methodology Approach
Source: Plants (Basel). 2026 Mar 19;15(6):951. doi: 10.3390/plants15060951 (PMC13030600; doi:10.3390/plants15060951)
Supplement: Supplementary file 1 [file plants-15-00951-s001.zip › plants-4151503-supplementary.pdf]

## Supplementary tables

**Table S1.** Pearson correlation coefficients (R) linking major bioactive markers to antioxidant capacity. Bold values indicate significant correlations (r > 0.80; p<0.05).

| Extraction fraction | | TEAC (Acetone) | TEAC (Hexane) | DPPH (Hexane) |
|---|---|---|---|---|
| **Lipophilic ($ScCO_2$)** | γ-Tocopherol | -0.69 | **0.91** | 0.80 |
| | Total flavonoids | **-0.88** | **1.00** | **0.96** |
| | SFA | -0.47 | **0.84** | **0.86** |
| | MUFA | -0.56 | **0.89** | **0.92** |
| | PUFA | 0.29 | 0.18 | 0.15 |
| | CLnA | 0.24 | 0.23 | 0.18 |
| | Punicic acid | 0.48 | -0.04 | -0.08 |
| | | **TEAC (Methanol)** | **FRAP (Water)** | **DPPH (Ethanol)** |
| **Hydrophilic (scW)** | Total phenolics | -0.51 | -0.64 | -0.58 |
| | Punicalagin α | **1.00** | **0.99** | **1.00** |
| | Punicalagin β | **0.99** | **1.00** | **1.00** |
| | Punicalin β | -0.68 | -0.79 | -0.73 |
| | Glucogallin | **-0.86** | **-0.92** | **-0.90** |
| | Gallic acid | -0.57 | -0.71 | -0.64 |
| | Ellagic acid | **0.86** | 0.76 | **0.82** |
| | Total flavonoids | -0.78 | **-0.88** | **-0.82** |
| | Soluble sugars | -0.49 | -0.61 | -0.56 |

**Table S2.** Two-way ANOVA summary for Figure 7: *Statistical significance was assessed by Two-way ANOVA, followed by the Holm–Sidak post-hoc test for multiple comparisons (n=3). Factors evaluated: "Phenol concentration" and "Formulation type" for dose-response data. The threshold for statistical significance was set at p < 0.05.*

| Experimental dataset | Source of variation | % of Total variation | *p* value |
|---|---|---|---|
| **HEK-293 cells** (Dose-response, 24-72h) | Formulation Type | 21.85 | < 0.0001 |
| | Phenol Concentration | 48.32 | < 0.0001 |
| | Interaction | 10.44 | 0.0012 |
| | Residual (Error) | 19.39 | |
| **MCF-7 cells** (Dose-response, 24-72h) | Formulation Type | 38.12 | < 0.0001 |
| | Phenol Concentration | 50.45 | < 0.0001 |
| | Interaction | 9.21 | < 0.0001 |
| | Residual (Error) | 2.22 | |

**Table S3.** Two-way ANOVA summary for Figure 7: *Statistical significance was assessed by Two-way ANOVA, followed by the Holm–Sidak post-hoc test for multiple comparisons (n=3). Factors evaluated: "Incubation time" and "Treatment type" for time-course data (Figure 8). The threshold for statistical significance was set at p < 0.05.*

| Experimental dataset | Source of variation | % of Total variation | *p* value |
|---|---|---|---|
| **Comparative Study** (Time-course, 0-72h) | Incubation Time | 42.60 | < 0.0001 |
| | Treatment Type | 35.18 | < 0.0001 |
| | Interaction | 16.22 | < 0.0001 |
| | Residual (Error) | 6.00 | |
